# Large-scale genetic profiling of hereditary ataxias in China: implications for a stepwise molecular diagnostic strategy

**DOI:** 10.1186/s40035-026-00584-y

**Published:** 2026-09-29

**Authors:** Jin-Yang Yu, Yi-Min Sun, Yi Dong, Jian-Jun Wu, Chen-Hao Zhu, Yi-Chu Du, Yi-Xin Zhang, Yue Zhang, Sheng-Mei Zou, Yin Ma, Hai-Lin Dong, Zhi-Ying Wu

**Affiliations:** 1 Department of Medical Genetics and Center for Rare Diseases, and Zhejiang Key Laboratory of Rare Diseases for Precision Medicine and Clinical Translation, Second Affiliated Hospital, Zhejiang University School of Medicine, Hangzhou, China; 2 Nanhu Brain-computer Interface Institute, Hangzhou, China; 3 Department of Neurology and National Research Center for Aging and Medicine & National Center for Neurological Disorders, State Key Laboratory of Medical Neurobiology, Huashan Hospital, Fudan University, Shanghai, China; 4 MOE Frontier Science Center for Brain Science and Brain-Machine Integration, School of Brain Science and Brain Medicine, Zhejiang University, Hangzhou, China

**Keywords:** Hereditary ataxias, Genetic landscape, Molecular diagnosis, Chinese population

## Abstract

**Background:**

Hereditary ataxias are clinically and genetically heterogeneous, which complicates clinical management, genetic counseling, and scientific research. Efficient molecular diagnosis is essential but requires molecular epidemiological considerations due to the variable genetic landscapes across regions and populations. In China, despite the established predominance of spinocerebellar ataxia type 3 (SCA3), comprehensive data on the genetic landscape of hereditary ataxias are limited and increasingly outdated, leaving insufficient evidence to support an epidemiology-informed molecular diagnostic strategy and its clinical implementation as a diagnostic algorithm.

**Methods:**

We retrospectively established a large, real-world, comprehensive cohort of hereditary ataxias (the HARMONY cohort) recruited from two medical centers in the Yangtze River Delta region of China (2008–2024) for genetic profiling. Molecular diagnoses were categorized into four classifications (‘Definitive’, ‘Probable’, ‘Unclear’, ‘Undiagnosed’) by integrating previously identified causative variants with results from systematic genetic testing in etiologically undetermined probands, and the associated variants were mapped onto genetic landscapes.

**Results:**

A total of 2096 probands were included in the HARMONY cohort, achieving a molecular diagnostic yield of 84.9%, with 1715 (81.8%) ‘Definitive’ and 65 (3.1%) ‘Probable’ positive diagnoses, alongside 87 (4.2%) ‘Unclear’ and 229 (10.9%) ‘Undiagnosed’ cases. While the genetic landscape was dominated by variants underlying SCA3 (1249; 59.6%), with SCA2 (150; 7.2%), SCA1 (139; 6.6%), SCA6 (51; 2.4%), putative SCA36 (36; 1.7%), and SCA12 (35; 1.7%) constituting the major subtypes, the genetic profiling revealed a long tail of rare genotypes. Onset age distributions were characterized for genotypes with ≥ 5 probands. No probands were found to carry expansions in *ZFHX3*, *BEAN1*, *DAB1*, *THAP11*, or *FXN*.

**Conclusions:**

Genetic profiling of the HARMONY cohort revealed a skewed genetic landscape dominated by a few common repeat-expansion subtypes, alongside a markedly heterogeneous long tail of rare etiologies. The holistic profile supports a stepwise molecular diagnostic strategy, under which we propose a YRD (Yangtze River Delta)-oriented candidate diagnostic algorithm. This study provides an evidence base for early molecular diagnosis, prioritization of therapeutic development, and adaptive design of diagnostic tools for hereditary ataxias.

**Supplementary Information:**

The online version contains supplementary material available at https://doi.org/10.1186/s40035-026-00584-y.

## Background

Hereditary ataxias (HAs) represent a group of genetic neurodegenerative disorders characterized by progressive movement incoordination, with primary clinical manifestations including gait imbalance, dysmetria, dysarthria, and oculomotor abnormalities [1]. As a core identifying feature of HA cases, ataxia may manifest concurrently with other neurological symptoms and occur at any age. This inevitably leads to a coexistence of phenotypic overlap and diversity both between HAs and other ataxia-related disorders and among various subtypes of HAs themselves, highlighting the clinical complexity and heterogeneity [2–4]. While acquired etiologies and non-hereditary degenerative conditions, comprising multiple system atrophy cerebellar type (MSA-C) and sporadic adult-onset ataxia of unknown etiology, account for a subset of ataxia cases in general populations, the definitive diagnosis of HAs necessitates meticulous exclusion of these mimics followed by molecular confirmation through methodical genetic testing [5, 6]. However, this analysis task is further compounded by the marked genetic heterogeneity of HAs. The disease group encompasses diverse classes of genetic variants, including tandem repeat expansions (TREs), conventional variants, and structural variants, such as copy number variants (CNVs), across more than a hundred documented disease-associated genes spanning autosomal-dominant (AD), autosomal-recessive (AR), X-linked, and mitochondrial inheritance patterns [7–10]. Due to the scarcity of phenotypic signatures with high sensitivity and specificity for particular causative genes, such complexity often generates substantial challenges and uncertainty in clinical practice, hindering effective clinical management, familial genetic counseling and scientific research for HA patients [11–13].

To address this dilemma, a stepwise or tiered molecular diagnostic strategy has been widely adopted: in the absence of additional suggestive clinical findings or clues from family history, prevalent TREs are first tested by PCR-based methods according to the presumed inheritance pattern, with rarer-TRE screening and next-generation sequencing (NGS) reserved for patients yielding negative initial results [8, 9, 14, 15]. This strategy essentially represents a cost-effective compromise with considerations from molecular epidemiology: until a single assay can economically resolve all genomic variant classes in routine clinical practice, the stepwise design leverages a skewed genetic landscape of HAs by prioritizing high-frequency etiologies to triage patients efficiently. An epidemiology-informed strategy achieves its clinical implementation through an efficient molecular diagnostic algorithm, which is essential for the widespread adoption of diagnostic advances, shortening the patient’s odyssey and raising yields. Yet both require adaptation across regions and populations, as the underlying genetic landscapes of HAs differ in subtype distribution, reflecting genetic background, founder effects, and other factors [11]. Therefore, genetic profiling studies of large-scale, context-specific HA cohorts are required to inform a guiding diagnostic strategy and its corresponding algorithm, by delineating comprehensive genetic landscapes aligned with the latest advances [12].

In China, HAs demonstrate a unique genetic landscape distinct from those observed in global cohorts, with spinocerebellar ataxia type 3 (SCA3) predominating among dominant ataxias, followed by SCA2 and SCA1. This is in notable contrast to the prevalence of *CACNA1A*-related ataxias in the UK, SCA1/2 in Italy, and SCA27B/3 in Germany and France [6, 11, 16]. The molecular epidemiology of HAs in the Chinese population has been characterized through a limited number of cohort studies, along with many case reports and case series focusing on specific subtypes [9, 11, 17–19]. Moreover, the few available Chinese cohort studies have either focused on specific modes of inheritance or involved modest sample sizes. As a result, they are insufficient to provide a comprehensive and in-depth depiction of the genetic landscape across the broader Chinese population, failing to rationalize a diagnostic strategy grounded in molecular epidemiology and its translation into an efficient algorithm. Beyond this, the genetic basis of HAs is rapidly expanding, with novel genes or pathogenic mechanisms, such as *RFC1*, GAA-*FGF14*, *THAP11* and *ZFHX3*, being identified through bioinformatic innovations and long-read sequencing [8, 20–23]. Mounting evidence also indicates a role for digenic interactions (e.g. *STUB1* with expansions in *TBP* or *ATXN8*/*ATXN8OS*) in modifying disease penetrance and expressivity [24, 25]. Despite the advances, the prevalence of these emerging genetic etiologies in the Chinese population remains poorly defined, underscoring the need for updated data.

Here, we introduce a large HA cohort established for genetic profiling under our novel research project, Hereditary Ataxia Research on Multi-Omics and Neuroclinical Insights in the Yangtze Delta (HARMONY, NCT07092358). The HARMONY project involves two leading medical centers in the Yangtze River Delta (YRD) region of China (including Shanghai, Jiangsu, Zhejiang, and Anhui): the Second Affiliated Hospital of Zhejiang University School of Medicine (SAHZU) and Huashan Hospital, Fudan University (HSFU). We aim to delineate a comprehensive genetic landscape of HAs encountered at the two centers in the YRD, propose a rationalized molecular diagnostic strategy together with an YRD-oriented candidate diagnostic algorithm, and contribute to the global understanding of this complex group of disorders.

## Methods

### Establishment of the HARMONY cohort

The HARMONY project is a two-center observational study designed to investigate the associations between multi-omics profiles and clinical phenotypes among HA patients primarily from the YRD region of China. We conducted a retrospective cross-sectional data collection from probands clinically or molecularly diagnosed with HAs at HSFU (Shanghai, January 2008–December 2024) and SAHZU (Hangzhou, Zhejiang, January 2015–December 2024). Recruitment occurred at the Departments of Neurology (HSFU, SAHZU) and Medical Genetics (SAHZU), which accepted suspected ataxia cases irrespective of age, covering both pediatric and adult populations. Demographic, clinical, and genetic data were recorded. Disease severity was graded using the Spinocerebellar Degeneration Functional Score (SDFS), a simple and rapidly applicable scale that allowed broad and representative coverage of the cohort, from 2010 onward [26, 27]. Family history was collected through interviews with probands and relatives, specifically inquiring about the health and age of parents and siblings. Cases were classified as familial if recurrence was observed in relatives, or sporadic if isolated. Vertical transmission indicated AD inheritance, whereas affected siblings or parental consanguinity suggested AR etiology. Archival peripheral blood specimens or extracted DNA from patients and available family members, previously stored in the biobanks at HSFU and SAHZU, were retrieved for genetic analysis. To eliminate duplicates, patient records were cross-referenced between the two centers before being fully anonymized with a project-specific identifier for subsequent analysis.

The clinical eligibility of each proband for HA diagnosis was independently reviewed and confirmed by at least two senior neurologists with expertise in movement disorders. Final enrollment was based on inclusion criteria that comprised three clinical items and one genetic item: (i) progressive ataxia as a predominant or consistent feature without coexisting cerebrovascular disease, brain tumors, or severe systemic illnesses; (ii) no clinical, neuroimaging, or laboratory evidence suggesting acquired ataxias, including chronic intoxication, nutritional deficiencies, immune-mediated inflammation, infections, acute injury, stroke, or space-occupying disorders (details in Additional file 1: Text S1) [5, 6]; (iii) for late-onset (≥ 30 years) ataxia patients with neither family history nor identified causative variants, the disease duration should exceed three years without prominent progressive autonomic dysfunction or other features suggestive of MSA-C [21, 28, 29]; and (iv) the causative genes identified in patients should fall within the scope of HAs, which is defined based on the consensus genetic classification synthesized from the Movement Disorder Society (MDS) Task Force on Classification and Nomenclature of Genetic Movement Disorders [13, 30] and the Society for Research on the Cerebellum and Ataxias (SRCA) Task Force [31], as well as the latest genetic advances. Specifically, the eligible genes were required to be either designated by MDS with an ‘ATX’ prefix or classified as ‘Primary’ by SRCA, while genes categorized by SRCA under ‘other metabolic or complex autosomal recessive disorders’ were excluded. The complete list of eligible genes for HAs is provided in Additional file 1: Table S1. No additional criteria, such as sex, age, ethnicity, family history, or prior genetic testing, were applied in patient recruitment.

### Management of molecular diagnosis

To systematically manage the molecular diagnoses of patients in the HARMONY cohort and retain candidate variants with limited evidence for further consideration, we categorized the molecular diagnoses using a tiered classification system, which includes four levels: ‘Definitive’, ‘Probable’, ‘Unclear’, and ‘Undiagnosed’, through integrated assessment of variant pathogenicity, gene-phenotype correlation, and established mechanistic alignment (Table 1).

**Table 1:** Molecular diagnosis classification and interpretation for the HARMONY cohort

Classification	Interpretation
Definitive	1. Identification of the following evident deleterious variant types with typical gene-phenotype correlation (required for both alleles in compound heterozygotes): a) Conventional variants (SNVs & indels) or CNVs classified as P or LP according to the ACMG guidelines; b) Repeat expansions reaching full-penetrance length thresholds with pathogenic motifs and configurations AND 2. Alignment of genetic findings with established pathogenesis and inheritance
Probable	While failing the above criteria: 1. Identification of the following possible deleterious variant types with typical gene-phenotype correlation (required for both alleles to meet or exceed this threshold in compound heterozygotes): a) Conventional variants classified as VUS exhibiting PM2 plus ≥ 1 additional pathogenic criterion (PP3 required for missense/splicing) or CNVs scoring ≥ 0.45, in the absence of benign evidence; b) Repeat expansions falling within reduced-penetrance length ranges with pathogenic motifs and configurations AND 2. Alignment of genetic findings with established pathogenesis and inheritance
Unclear	While failing the above criteria: 1. Identification of the following ambiguous deleterious variant types with typical gene-phenotype correlation and alignment with established pathogenesis and inheritance (required for both alleles to meet or exceed this threshold in compound heterozygotes): a) Conventional variants classified as VUS exhibiting PM2 but lacking additional pathogenic criteria (with prerequisite PP3 unmet for missense/splicing) or CNVs scoring 0.15–0.44, in the absence of benign evidence; b) Repeat expansions experimentally confirmed but with indeterminate length, motif, or configuration OR 2. Identification of the above evident or possible deleterious variant types with compatible gene-phenotype correlation and alignment with established pathogenesis and inheritance; OR 3. Identification of the above evident or possible deleterious variant types with typical gene-phenotype correlation but showing poor alignment with established pathogenesis and inheritance
Undiagnosed	Absence of candidate genetic findings

*ACMG* the American College of Medical Genetics and Genomics, *CNV* copy number variant, *LP* likely pathogenic, *P* pathogenic, *SNV* single nucleotide variant, *VUS* variant of uncertain significance

‘Definitive’ and ‘Probable’ levels were designated as positive molecular diagnoses, representing the diagnostic yield. Relevant variants underlying the molecular diagnoses of ‘Definitive’, ‘Probable’, and ‘Unclear’ were collectively termed ‘genetic findings’. Among these, conventional variants and CNVs were designated as ‘known’ if listed as disease-causing in the Human Gene Mutation Database (HGMD, Professional 2025.1), and ‘novel’ otherwise. For the ClinVar database, unreported variants were similarly designated as ‘novel’, whereas reported variants were further annotated with their specific rating (e.g., Pathogenic, Likely pathogenic, Variant of uncertain significance, or Conflicting classifications of pathogenicity).

Specifically, given that the massive disease-causing expansions in *NOP56* (SCA36) and *ATXN10* (SCA10) preclude precise sizing by PCR-based methods, leaving pathogenic threshold attainment unknown, suggestive results of an expanded allele were annotated as ‘putative’ and classified as ‘Unclear’ (see below for technical details). Furthermore, certain genotypes for which the pathogenesis or inheritance remains insufficiently established were also categorized as ‘Unclear’. These included isolated occurrences of intermediate *TBP* alleles (41–48 repeats, denoted as ‘*TBP*-i’) or *ATXN8*/*ATXN8OS* (hereafter, *ATXN8*) expansions, both of which exhibit reduced penetrance or high population frequency, suggesting possible roles as susceptibility factors rather than established Mendelian causes [32–34]. Similarly, genotypes involving monoallelic *STUB1* variants were categorized as ‘Unclear’ due to the uncertain dominant mechanism of SCA48 (distinct from the established recessive pathogenicity) and their frequent co-occurrence with *TBP*-i or *ATXN8* expansions [35]. In addition, we used notations such as ‘GAA-*FGF14*’ vs. ‘*FGF14*’ and ‘CAG-*CACNA1A*’ vs. ‘*CACNA1A*’ to denote different pathogenic mechanisms (TREs vs. conventional variants) within the same gene.

### Genetic testing and analysis

**Fig. 1:**
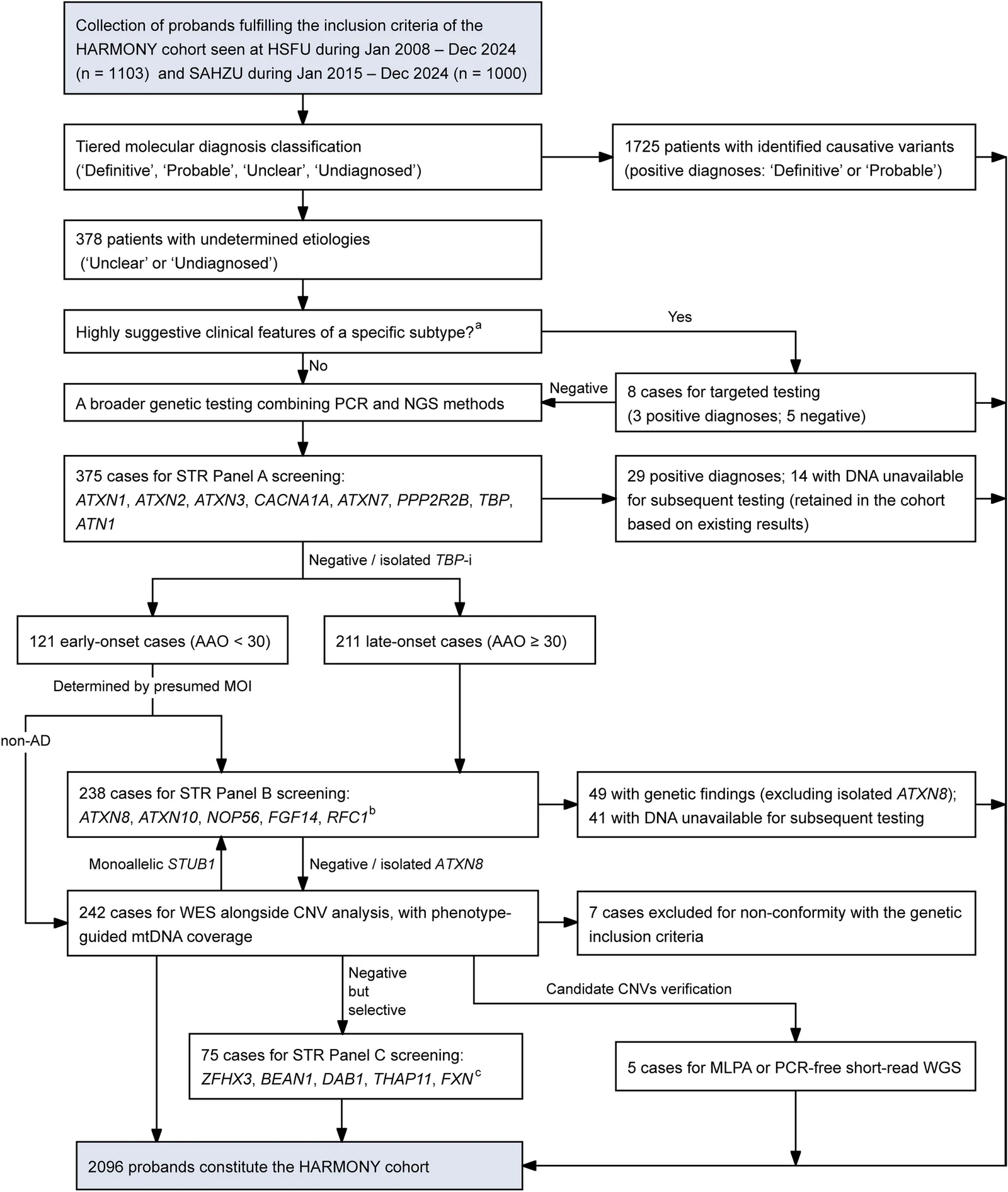
Study profile and genetic testing workflow. ^a^For example, neuropathy, vestibular dysfunction, chronic cough, and an onset age over 35 years indicate *RFC1* locus screening; macular degeneration, progressive visual loss, and an AD family history indicate *ATXN7* locus screening; seizures, myopathy, sensorineural hearing impairment, and stroke-like hyperintensities indicate mtDNA sequencing. ^b^*RFC1* screening will be included in Panel B or performed exclusively when the mode of inheritance (MOI) is suggestive of or strongly indicates an AR pattern, respectively. ^c^*FXN* screening will be included in Panel C or performed exclusively when the MOI is suggestive of or strongly indicates an AR pattern, respectively. AAO: age at onset; AD: autosomal dominant; AR: autosomal recessive; CNV: copy number variant; HARMONY: Hereditary Ataxia Research on Multi-Omics and Neuroclinical Insights in the Yangtze Delta; HSFU: Huashan Hospital, Fudan University; MLPA: multiplex ligation-dependent probe amplification; mtDNA: mitochondrial DNA; NGS: next-generation sequencing; PCR: polymerase chain reaction; SAHZU: the Second Affiliated Hospital of Zhejiang University School of Medicine; STR: short tandem repeat; WES: whole exome sequencing; WGS: whole genome sequencing

Genomic DNA was extracted from peripheral blood leukocytes of participants using the QIAamp Blood Mini Kit or Puregene Blood Kit (Qiagen, Hilden, Germany) depending on the required DNA fragment size for downstream applications, according to the manufacturer’s standard protocol. Upon enrollment into the cohort, most patients had received various genetic assessments driven by clinical discretion and the accessibility of testing approaches at the time, ranging from targeted TRE analysis to NGS-based targeted gene panel sequencing or whole exome sequencing (WES) with variable mitochondrial DNA (mtDNA) coverage. Probands with previously identified causative variants meeting ‘Definitive’ or ‘Probable’ criteria were directly included. In contrast, those without prior genetic testing or with inconclusive prior results (‘Unclear’ or ‘Undiagnosed’) underwent a systematic genetic testing workflow to complete their genetic assessments (Fig. 1).

Probands with undetermined etiologies were first assessed for highly suggestive clinical features warranting targeted testing (e.g., ataxia with myoclonus, epilepsy, or choreoathetosis with an AD family history, suggestive of dentatorubral-pallidoluysian atrophy). In the absence of such findings or following negative targeted testing, probands proceeded to a broader investigation structured around three sequential short tandem repeat (STR) panels curated based on previously reported frequency and research priorities. The investigation commenced with screening using STR Panel A (*ATXN1*, *ATXN2*, *ATXN3*, *CACNA1A*, *ATXN7*, *PPP2R2B*, *TBP*, and *ATN1*) for common CAG TREs. When Panel A testing was negative or revealed an isolated *TBP*-i, downstream analysis was guided by an age at onset (AAO) cut-off of 30 years, dividing the probands into early-onset (< 30 years) and late-onset (≥ 30 years) groups. Among the late-onset group, we proceeded with STR Panel B (*ATXN8*, *ATXN10*, *NOP56*, *FGF14*, with *RFC1* added for sporadic cases or tested alone in AR cases). Probands with negative results or isolated *ATXN8* expansions from Panel B underwent WES alongside CNV analysis, with phenotype-guided mtDNA coverage, to explore non-TRE variants. For early-onset probands, the testing sequence was stratified by the presumed mode of inheritance: non-AD cases proceeded directly to WES, with reflex Panel B screening upon detection of candidate monoallelic *STUB1* variants, whereas others followed the routine Panel B-to-WES trajectory. If no genetic findings were identified after the above testing in both groups, further investigation with a third research-oriented STR panel (STR Panel C: *ZFHX3*, *BEAN1*, *DAB1, THAP11*, and *FXN*) was performed for a subset of probands according to the inheritance patterns, with AR cases limited to *FXN* testing and sporadic cases undergoing the full panel.

#### TRE screening

PCR-based assays for STR Panel A were performed as previously described [10]. For precise determination of repeat lengths, fluorescent fragment analysis was conducted on an ABI 3730xL Genetic Analyzer using SIZE600 (XiangYin Biotechnology, Hangzhou, China) as a size standard, with data processed by GeneMarker software for fragment sizing and allele calling. Fluorescent tethered repeat-primed PCR was employed for loci in which large repeat expansions, if present, are refractory to flanking PCR, or for those requiring detailed internal motif analysis (e.g., *ATXN10*, *NOP56*, *RFC1*, *FGF14*, *BEAN1*, and *DAB1*). The amplification products were subsequently analysed by capillary electrophoresis on the same instrument, where a characteristic ladder or sawtooth peak pattern suggested the presence of an allele with a specific repeat expansion. PCR amplifications for STR Panels B and C were carried out as described in Additional file 1: Text S2.

#### Next-generation sequencing and analysis

The WES workflow, including library preparation, sequencing, quality control, alignment, annotation, variant filtering, and ExomeDepth-based CNV analysis, was carried out as previously described [9]. Pathogenicity of missense or splicing variants was further evaluated using a suite of in silico prediction tools, including REVEL, AlphaMissense, VARITY, PrimateAI, and SpliceAI. Candidate conventional variants and CNVs were classified according to the guidelines issued by the American College of Medical Genetics and Genomics [36, 37], with conventional variants validated by Sanger sequencing and CNVs confirmed by either ligation-dependent probe amplification (MLPA) or PCR-free whole genome sequencing (WGS). The detailed methodology for the WGS pipeline is provided in Additional file 1: Text S3. In the absence of parental DNA, allele-specific long-range PCR spanning the variants, followed by Sanger sequencing, was performed to resolve variant phasing where feasible.

### Statistical analysis

Categorical variables were compared using the chi-square test; continuous and ordinal variables were compared using the Wilcoxon rank-sum test for two-group comparisons or the Kruskal–Wallis test for multi-group comparisons. Post-hoc pairwise comparisons with Benjamini–Hochberg correction (*P*_BH_) were performed where applicable. The combined effect of AAO and family history on the detection rate of genetic findings was assessed using logistic regression, with results presented as odds ratios (ORs) and 95% confidence intervals (CIs). Overall similarity between groups in their multi-category frequency profiles was quantified using the Bray–Curtis similarity index (BCSI, range: 0–1, where 1 indicates identical composition) and the Jensen–Shannon divergence (JSD, computed using log base 2, range: 0–1, where 0 indicates identical composition) [38, 39]. Analyses were performed using R software (version 4.4.3), and a *P*-value < 0.05 was considered statistically significant.

## Results

### Overview of the HARMONY cohort

**Fig. 2:**
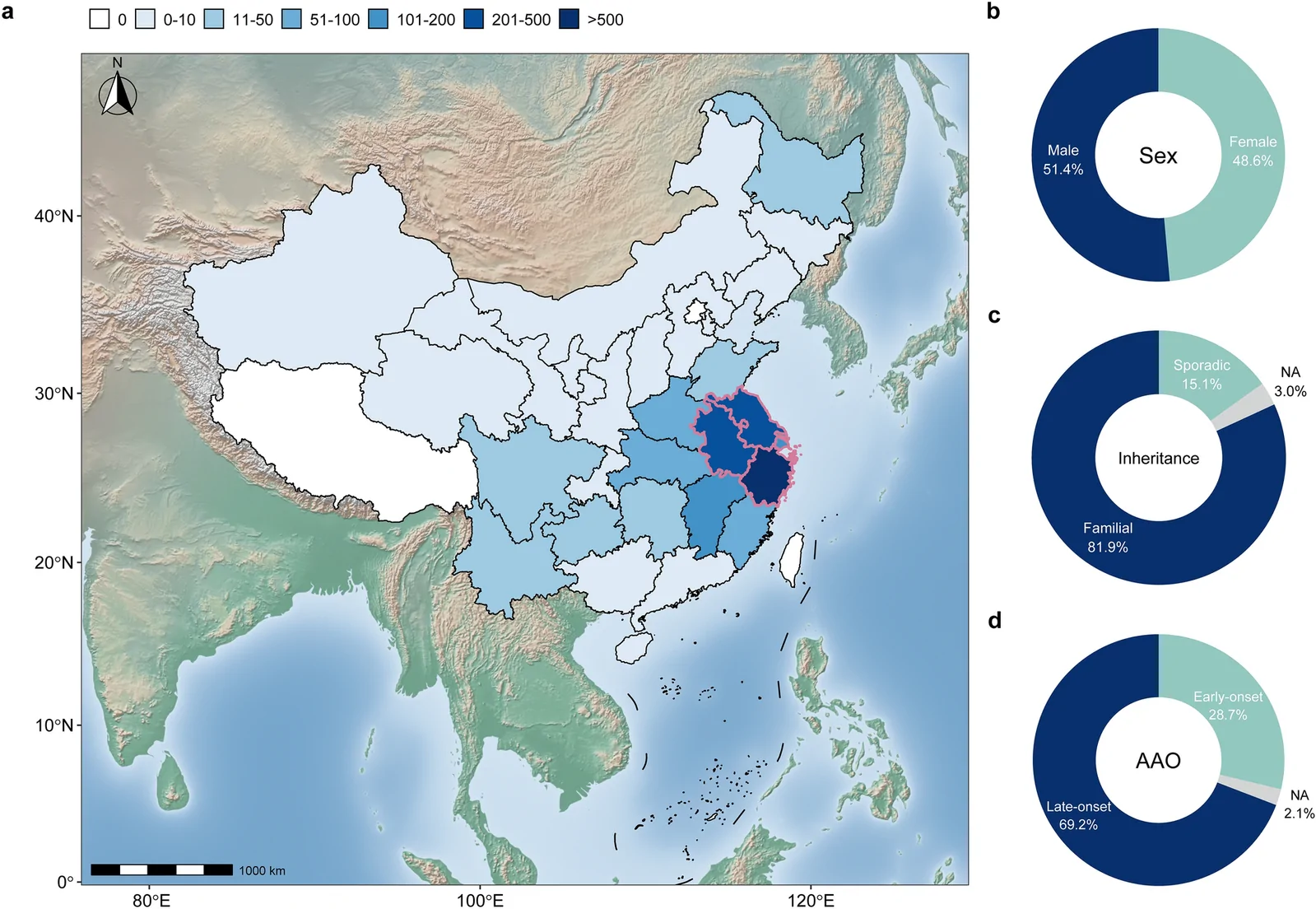
Demographic and clinical characteristics of probands in the HARMONY cohort. **a** Geographical distribution of 2068 probands from the HARMONY cohort. The intensity of the color reflects the number of patients. The top four regions are Zhejiang, Jiangsu, Anhui, and Shanghai, all of which are part of the Yangtze River Delta in China (outlined in pink). **b–d** Distribution profile of the HARMONY cohort across sex (**b**), mode of inheritance (**c**), and age at onset (**d**), classified as early- (< 30 years) or late-onset (≥ 30 years). NA: not available

A total of 2103 probands meeting the inclusion criteria were initially enrolled. Of these, 1725 harbored previously identified causative variants supporting ‘Definitive’ or ‘Probable’ molecular diagnoses, while the remaining 378 were etiologically undetermined and provisionally classified as ‘Unclear’ or ‘Undiagnosed’. Following systematic genetic testing of the latter group, DNA samples became unavailable in 14 cases after Panel A screening and in another 41 cases after Panel B. These probands were retained in the cohort based on existing results. Seven probands were excluded for non-conformity with the genetic inclusion criteria, as the subsequently identified candidate variants involved genes outside the curated scope of HAs (Fig. 1, Table S2). Ultimately, 2096 probands with molecularly confirmed or clinically suspected HAs were enrolled in this disease cohort (997 from SAHZU and 1099 probands from HSFU). Among the 2068 participants with documented place of origin, individuals were geographically distributed across 29 provincial-level regions in China, with a majority in Zhejiang (851; 41.2%), Jiangsu (315; 15.2%), Anhui (244; 11.8%), and Shanghai (160; 7.7%), resulting in a predominant concentration (1570; 75.9%) in the YRD region (Fig. 2a, Table S3).

As detailed in Table 2, the cohort showed a balanced sex ratio (1078 males; 51.4%) (Fig. 2b). Familial cases constituted the majority (1716; 81.9%), followed by sporadic cases (317; 15.1%) and missing data (63; 3.0%) (Fig. 2c), with the SAHZU group showing more sporadic cases compared to the HSFU group (22.5% vs. 8.5%; *P* < 0.001). Onset age was recorded for 2052 probands (median = 38 years, range = 0–77 years): 28.7% (*n* = 602) were classified as early-onset (< 30 years), 69.2% (*n* = 1,450) as late-onset (≥ 30 years), and 2.1% (*n* = 44) had an unknown AAO (Fig. 2d). Notably, the proportions of early- and late-onset cases were comparable between the SAHZU and HSFU groups (*P* > 0.05), despite a significantly earlier median AAO in the HSFU group (39 vs. 36 years; *P* = 0.004). SAHZU participants presented with a higher median age at examination (45 vs. 41 years; *P* < 0.001) and a longer median disease duration (5 vs. 4 years; *P* < 0.001). Conversely, the HSFU group had a significantly higher proportion of patients with prior positive molecular diagnoses (76.6% vs. 87.4%; *P* < 0.001). SDFS at last examination, available for 1846 (88.1%) probands, predominantly fell at level 3 in both centers (median = 3, inter-quartile range [IQR]: 3–3; *P* < 0.001). The proportion of probands originating from the YRD was comparable between the two centers (75.9% vs. 76.0%; *P* = 0.97), with variation in the contributing provinces: SAHZU drew predominantly from Zhejiang (636; 64.2%), while HSFU recruited mainly from Jiangsu (285; 26.5%), Zhejiang (215; 20.0%), and Anhui (160; 14.9%), rather than local Shanghai (158; 14.7%).

**Table 2:** General characteristics of the HARMONY cohort

	All Probands (*n* = 2096)	SAHZU (*n* = 997)	HSFU (*n* = 1099)	*P*^a^
Sex				0.051
Male	1078 (51.4%)	490 (49.1%)	588 (53.5%)	
Female	1018 (48.6%)	507 (50.9%)	511 (46.5%)	
Family history				**< 0.001**
Familial	1716 (81.9%)	756 (75.8%)	960 (87.4%)	**< 0.001**
Sporadic	317 (15.1%)	224 (22.5%)	91 (8.5%)	**< 0.001**
Unknown	63 (3.0%)	17 (1.7%)	46 (4.2%)	**0.001**
Age at onset				0.052
Early-onset (< 30 years)	602 (28.7%)	286 (28.7%)	316 (28.8%)	1.000
Late-onset (≥ 30 years)	1450 (69.2%)	698 (70.0%)	752 (68.4%)	0.461
Unknown	44 (2.1%)	13 (1.3%)	31 (2.8%)	**0.023**
Median year [IQR]^b^	38 [27–47]	39 [27–49]	36 [28–46]	**0.004**
Age at examination (Median year [IQR])^c^	43 [32–54]	45 [33–55]	41 [32–52]	**< 0.001**
Disease duration (Median year [IQR])^d^	4 [2–8]	5 [3–9]	4 [2–7]	**< 0.001**
Prior positive molecular diagnosis	1725 (82.3%)	764 (76.6%)	961 (87.4%)	**< 0.001**
SDFS at last examination (Median score [IQR])^e^	3 [3–3]	3 [3–3]	3 [3–3]	**< 0.001**
The YRD origin^f^	1570 (75.9%)	752 (75.9%)	818 (76.0%)	0.97

^a^Difference between SAHZU and HSFU groups, with statistically significant *P* values shown in bold

^b^Calculated based on 2052 probands with age at onset documented

^c^Calculated based on 2089 probands with age at examination documented

^d^Calculated based on 2047 probands with both age at onset and age at examination documented

^e^Calculated based on 1846 probands with SDFS

^f^Calculated based on 2068 probands with place of origin documented

*HSFU* Huashan Hospital, Fudan University, *SAHZU* the Second Affiliated Hospital of Zhejiang University School of Medicine, *SDFS* Spinocerebellar Degeneration Functional Score, *YRD* Yangtze River Delta

### Molecular genetic results

**Fig. 3:**
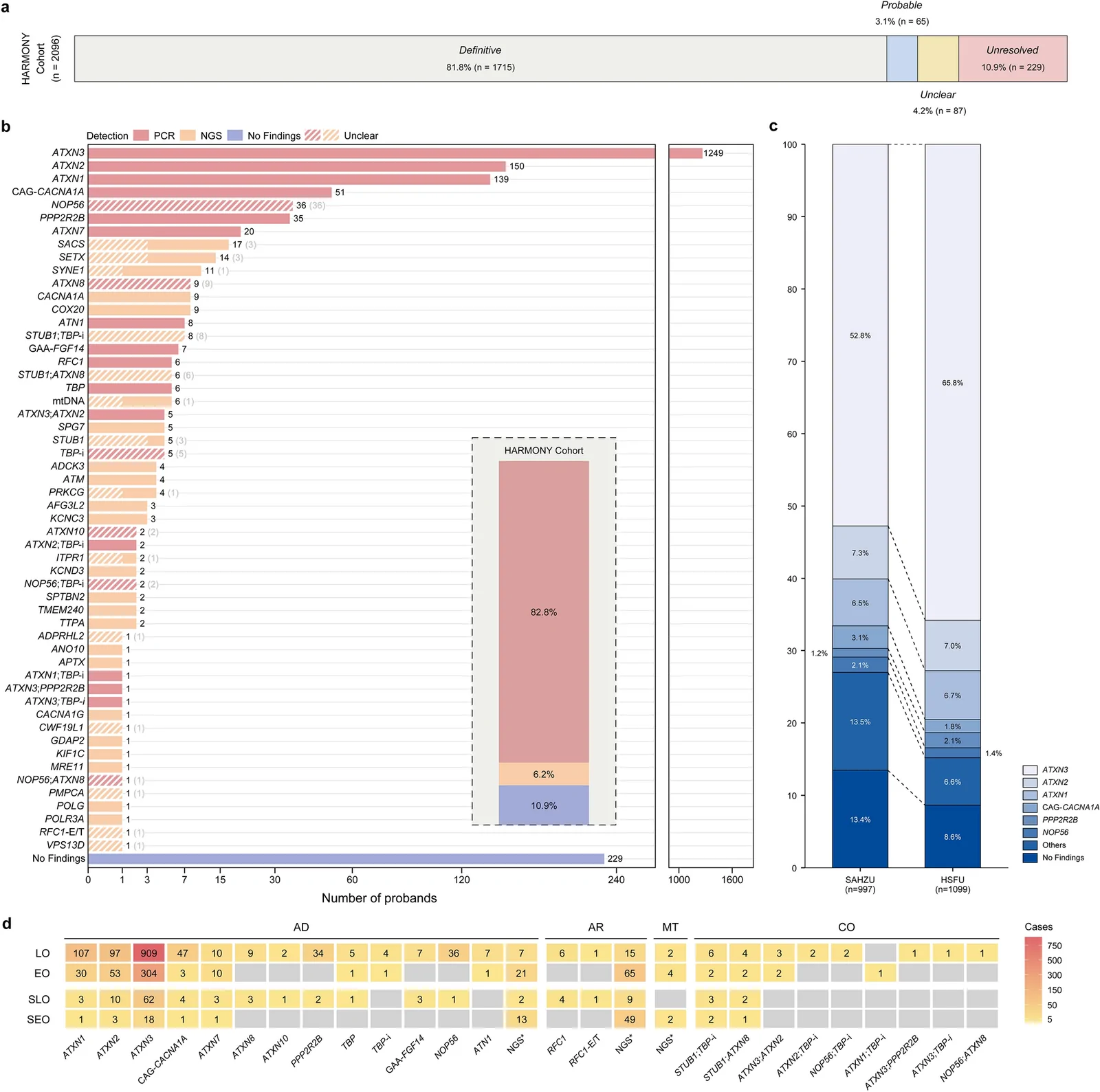
Landscapes of genetic findings in the HARMONY cohort. **a** Distribution of molecular diagnosis classifications within the HARMONY cohort. The ‘Definitive’, ‘Probable’, and ‘Unclear’ levels comprise all identified HA-associated genetic findings. **b** Breakdown of genetic findings by genotype. For better display, the x-axis has been transformed to grow quadratically and has a break from 275 to 900. Genotypes involving two HA genes are denoted by semicolon-separated gene names. CAG-*CACNA1A* refers to SCA6 caused by CAG repeat expansion (RE) in *CACNA1A*, which is distinct from conventional variants in the same gene (genotype *CACNA1A*). Likewise, GAA-*FGF14* refers to SCA27B caused by GAA RE in *FGF14*, which is distinct from conventional variants in *FGF14* (genotype *FGF14*). *TBP*-i denotes an intermediate RE in *TBP*. *RFC1*-E/T denotes the co-occurrence of a pathogenic RE (E) and a truncating variant (T) in *RFC1*. White diagonal hatching marks cases with an ‘Unclear’ molecular diagnosis in each genotype, while grey numbers in parentheses denote their counts. The subplot represents the distribution of cases with genetic findings detected solely by PCR (red), cases involving NGS (orange), and cases without genetic findings (No Findings, blue) in the cohort. **c** Comparison of case distributions between the two medical centers across the six most prevalent genetic findings, the other genetic findings (Others), and cases without genetic findings (No Findings). **d** Landscape of genetic findings stratified by clinical context (AAO and family history) and gene-defined inheritance/co-occurrence categories. ^a^Genetic findings identified solely by NGS are grouped under ‘NGS’. CO: co-occurrence; EO: early-onset; LO: late-onset; MT: mitochondrial; SEO: sporadic early-onset; SLO: sporadic late-onset

The molecular diagnostic yield was 84.9% (*n* = 1780), representing positive molecular diagnoses that included 1715 (81.8%) ‘Definitive’ and 65 (3.1%) ‘Probable’ cases. Genetic findings led to an ‘Unclear’ diagnosis in 4.2% (*n* = 87) cases and 10.9% (*n* = 229) cases were classified as ‘Undiagnosed’ without any candidate variants (Fig. 3a). Involving the ‘Definitive’, ‘Probable’, and ‘Unclear’ diagnoses of the probands, we aggregated all genetic findings by genotypes labeled with different pathogenic mechanisms to create a comprehensive genetic landscape (Fig. 3b). Among all probands, genetic findings detectable by PCR alone accounted for 82.8%, while NGS further closed the gap by 6.2%. While the genetic landscape was dominated by variants underlying SCA3 (1249; 59.6%), with SCA2 (150; 7.2%), SCA1 (139; 6.6%), SCA6 (51; 2.4%), putative SCA36 (36; 1.7%), and SCA12 (35; 1.7%) constituting the major subtypes, the genetic profiling revealed a long tail of rare genotypes: 48 of 54 genotypes (88.9%) each accounted for less than 1% of the cohort.

We examined whether the genotype frequency profiles varied geographically using BCSI and JSD metrics rather than hypothesis testing, which is poorly suited for sparse multi-category comparisons. Genotype frequencies were largely congruent between probands from within and outside the YRD (BCSI = 0.886; JSD = 0.035), with subtle differences driven primarily by a higher proportion of SCA3 in the YRD and higher proportions of SCA1, SCA6, and putative SCA36 in the non-YRD (Table S4). Within the YRD, pairwise comparisons among the four contributing provinces showed broadly comparable mutual similarity (BCSI range: 0.836–0.916, JSD range: 0.036–0.100; Table S5), with Jiangsu and Shanghai matching most closely and Anhui exhibiting the most distinct profile.

The detection rate of genetic findings differed significantly across the clinical contexts defined by onset age and family history (Table 3; *χ*^2^ = 232.0, *df* = 3, *P* < 0.001). Logistic regression revealed that a positive family history was associated with a higher detection rate, largely independent of onset age; rates were 93.8% in the familial late-onset subgroup (reference) and 91.9% in the familial early-onset subgroup (OR = 0.75, 95% CI [0.50, 1.15]; *P* = 0.18). Conversely, sporadic presentation was associated with a stepwise decline in detection rates, dropping to 71.7% in late-onset cases (OR = 0.17, 95% CI [0.11, 0.26]; *P* < 0.001) and further to 58.0% in early-onset cases (OR = 0.09, 95% CI [0.06, 0.13]; *P* < 0.001).

**Table 3:** Detection rate of genetic findings and logistic regression analysis by onset age and family history

Group	Total	Detected^a^	Undetected	Odds Ratio (95% CI)	*P*
Familial late-onset	1252	1174 (93.8%)	78 (6.2%)	Ref.	–
Sporadic late-onset	159	114 (71.7%)	45 (28.3%)	0.17 (0.11, 0.26)	**< 0.001**
Familial early-onset	431	396 (91.9%)	35 (8.1%)	0.75 (0.50, 1.15)	0.18
Sporadic early-onset	157	91 (58.0%)	66 (42.0%)	0.09 (0.06, 0.13)	**< 0.001**

^a^Genetic findings include variants underlying ‘Definitive’, ‘Probable’ and ‘Unclear’ molecular diagnoses

95% CI: 95% confidence interval for the odds ratio; Ref.: reference group

*P* values in bold indicate statistical significance

#### Predominance of repeat expansions

Repeat expansions were the predominant contributors to the positive molecular diagnoses, accounting for 1681 of 1780 cases (94.4%). Among these, SCA3, SCA2, SCA1, SCA6, and SCA12 were the five most common subtypes. Specifically, CAG repeat expansions in *ATXN3* alone explained the genetic etiology in 59.6% of the whole cohort, and the proportion cumulatively increased to 77.5% when SCA2, SCA1, SCA6, and SCA12 were included. Although probands with putative SCA36 were classified as ‘Unclear’ owing to indeterminate repeat expansion sizes by PCR-based testing, the detection of an abnormal expansion in *NOP56* was not rare (*n* = 39, including three cases with additional coexisting variants) and its monogenic genotype ranked fifth in frequency, accounting for 1.7% of all probands.

We compared the distribution of genetic findings between the two medical centers (Fig. 3c). The six most common genotypes in both groups were exclusively repeat expansions, collectively accounting for 73.0% of probands in SAHZU and 84.8% in HSFU. Beyond the top six genotypes, those remaining were grouped as “Others”, whereas “No Findings” (‘Undiagnosed’) was kept separate. While these aggregated molecular categories differed significantly between the two centers (*χ*^2^ = 59.6, df = 7, *P* < 0.001; Additional file 1: Table S6), quantitative similarity metrics still indicated largely congruent distributions (BCSI = 0.859; JSD = 0.021). Post-hoc testing showed that *ATXN3* repeat expansions were less common at SAHZU than at HSFU (52.8% vs. 65.8%; *P*_BH_ = 0.021). Conversely, the “Others” (13.5% vs. 6.6%; *P*_BH_ = 0.002) and the “No Findings” (13.4% vs. 8.6%; *P*_BH_ = 0.043) were more frequent at SAHZU. No significant differences were found for the other major genotypes (all *P*_BH_ > 0.05).

#### Diagnostic contribution of short-read sequencing

Conventional and structural variants detected by sequencing accounted for only 5.6% (*n* = 99) of the positive molecular diagnoses; however, their contribution surged to 63.5% after excluding the five most common confirmed repeat-expansion disorders. A total of 355 probands underwent targeted NGS, including 71 tested via targeted gene panel sequencing and 315 via WES with variable mtDNA coverage. Targeted panel sequencing yielded a positive diagnosis in 19.7% of cases (*n* = 14), a rate that rose to 22.5% (*n* = 16) when the genetic findings in ‘Unclear’ cases were included. In comparison, WES achieved a diagnostic yield of 27.0% (*n* = 85), which expanded to 36.5% (*n* = 115) with the inclusion of variants underlying ‘Unclear’. Of the 45 HA genes implicated across the cohort, NGS thus contributed to the positive diagnoses for 28 (62.2%), expanding to 34 (75.6%) when ‘Unclear’ diagnoses were included. Within the overall genetic findings, the non-TRE variants identified through targeted NGS most frequently involved *STUB1* (*n* = 19), *SACS* (*n* = 17), *SETX* (*n* = 14), *SYNE1* (*n* = 11), *CACNA1A* (*n* = 9), and *COX20* (*n* = 9). In addition, one proband diagnosed with Cerebellar Ataxia, Neuropathy and Vestibular Areflexia Syndrome (CANVAS) carried a heterozygous AAGGG expansion in *RFC1* together with a truncating variant, denoted as *RFC1*-Expansion/Truncating (*RFC1*-E/T). Although consistent with a reported pathogenic mechanism [40], this case was classified as ‘Unclear’ for the lack of co-segregation confirmation. Our previously unreported genetic findings attributed to conventional and structural variants identified by targeted NGS are summarized in Table S7.

In our previous study [9], we performed WES on 54 probands with suspected AR cerebellar ataxia who were subsequently included in the HARMONY cohort. Using ExomeDepth-based CNV analysis, we identified a de novo microdeletion in *SACS* and uniparental disomy affecting *ADCK3*. During this expanded implementation of the systematic genetic testing workflow, we identified compound heterozygosity involving a pathogenic single-nucleotide variant and an additional allelic pathogenic CNV within a single recessive gene in four probands, as well as a homozygous intragenic deletion in another proband. The involved genes were *MRE11* (*n* = 1), *SETX* (*n* = 2), and *SPG7* (*n* = 2). A detailed molecular characterization of these five probands is provided in Fig. S1.

#### Co-occurrence of genetic findings

The HARMONY cohort also included 27 probands with co-occurring genetic findings, involving candidate variants in two genes. These combinations comprised either variants in two different TRE loci or a conventional variant coexisting with a TRE: *STUB1*; *TBP*-i (*n* = 8), *STUB1*; *ATXN8* (*n* = 6), *ATXN3*; *ATXN2* (*n* = 5), *ATXN2*; *TBP*-i (*n* = 2), *NOP56*; *TBP*-i (*n* = 2), *ATXN1*; *TBP*-i (*n* = 1), *ATXN3*; *PPP2R2B* (*n* = 1), *ATXN3*; *TBP*-i (*n* = 1), and *NOP56*; *ATXN8* (*n* = 1). Among these findings, the most prevalent genotypes included *STUB1*-related coexisting combinations and SCA3/SCA2 co-occurrences. Specifically, we identified candidate *STUB1* variants in 19 probands: three carried compound heterozygous variants, while the remaining 16 were carriers of monoallelic variants. Among these monoallelic cases, we identified 8 (50.0%) patients harboring additional *TBP*-i and 6 (37.5%) patients carrying coexisting expansions in *ATXN8*. Reciprocally, of the 13 individuals with isolated *TBP*-i, 12 underwent NGS (DNA unavailable in one case), 8 of whom carried rare *STUB1* variants; likewise, 14 of 15 individuals with isolated *ATXN8* expansions underwent NGS (DNA unavailable in one case), 6 of whom harbored rare *STUB1* variants. In particular, we classified both ‘*STUB1*; *TBP*-i*’* and ‘*STUB1*; *ATXN8’* genotypes as ‘Unclear’ due to the inconclusive evidence for *STUB1* variants as modifiers in these contexts.

### Clinical stratification and onset age distributions

Genetic findings were additionally mapped onto a landscape according to the clinical contexts (onset age and family history), based on the inheritance patterns of the associated genes, with the co-occurrence presented separately (Fig. 3d). The three most common SCAs (1, 2, and 3) remained prevalent across the subgroups, including the sporadic late-onset cases with potentially the greatest etiological heterogeneity. Nevertheless, the early-onset patients presented a different profile, characterized not only by the absence of SCA12, SCA27B, putative SCA36, and CANVAS, but also by a prominent proportion of non-TRE variants detected by NGS, especially in those without family history.

**Fig. 4:**
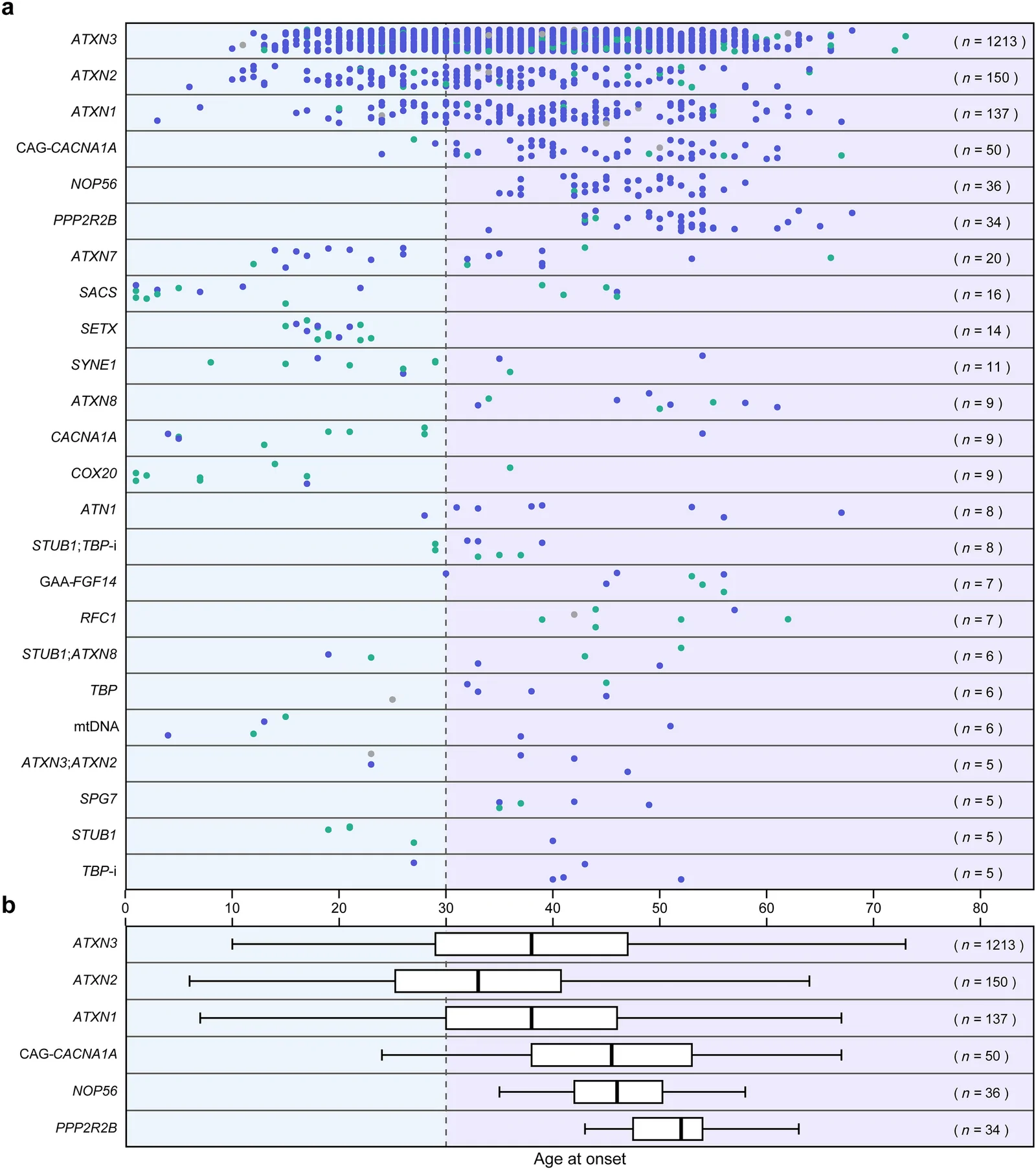
Onset age distribution of genotypes with five or more probands. **a** The ages at onset of probands are plotted as dots along the x-axis. Blue dots represent familial cases, green dots indicate sporadic cases, and unavailable data are shown as grey dots. The dashed line marks the 30-year threshold used to classify cases as early- or late-onset. **b** The box plot for genotypes, each comprising more than 30 probands. Each box represents the inter-quartile range (IQR), with the lower and upper boundaries corresponding to the 25th and 75th percentiles, respectively. The line inside the box is the median. The whiskers extend to the most extreme values within 1.5 times the IQR

Patients in the HARMONY cohort exhibited a wide range of onset age. Moreover, different AAO profiles emerged when analyzing each genotype. We summarized and visualized the AAO distributions for genotypes with five or more probands (Fig. 4a) and generated box plots for subgroups comprising more than 30 probands (Fig. 4b). Among the most common SCAs, SCA3, SCA2, and SCA1 showed broader AAO distributions. The median AAO was 38 years for *ATXN3* (range: 10–73; IQR: 29–47), 33 years for *ATXN2* (range: 6–64; IQR: 25–41), and 38 years for *ATXN1* (range: 3–67; IQR: 30–46). AAO distributions differed significantly among the three genotypes (Kruskal–Wallis *H* = 20.97, *P* < 0.001), with post-hoc Wilcoxon comparisons localizing the difference to SCA2, which showed earlier onset than both SCA1 and SCA3 (*P*_BH_ < 0.001 for both). In contrast, no significant difference was observed between SCA1 and SCA3 (*P*_BH_ = 0.72). SCA6, SCA12, and putative SCA36 exhibited a later onset tendency compared to SCA1, SCA2, and SCA3, with SCA12 and putative SCA36 exclusively presenting beyond the third decade, suggesting a reduced likelihood of anticipation in these subtypes. Notably, patients with genetic findings in *SETX* showed a remarkably uniform AAO, presenting around the age of 19. Conventional variants in *SACS*, *SYNE1*, and *CACNA1A* were also associated with an earlier AAO, typically before 30 years. In addition, patients with co-occurring *STUB1* and *TBP*-i variants exhibited a predominantly mid-adult onset, mainly between 30 and 40 years, whereas those with coexisting variants in *STUB1* and *ATXN8* presented with a broader AAO profile.

### STR Panel C screening in the ‘Undiagnosed’ probands

We screened a subset of the ‘Undiagnosed’ probands in the HARMONY cohort using STR Panel C to verify whether these subtypes are indeed rare in the Chinese population. Among the 229 ‘Undiagnosed’ probands (familial: 113, 49.3%; sporadic: 111, 48.5%; unknown: 5, 2.2%), we selected 75 individuals for stratified screening based on inheritance features. For the 32 probands presenting with apparent AD inheritance, we focused exclusively on screening for the expansions in *THAP11*, *ZFHX3*, *BEAN1*, and *DAB1*. In contrast, for the 10 probands with clear AR inheritance, testing was restricted to the *FXN* expansion. The remaining 33 sporadic probands were screened for both the AD-associated loci and *FXN*. However, no pathogenic expansions were identified at any of these tested loci across this subgroup.

## Discussion

Delineating the overall genetic landscape of HAs in a population is a time-consuming and systematically demanding task due to the remarkable intrinsic genetic heterogeneity of HAs. Given the limited data on the molecular epidemiology of HAs in the Chinese population, we established the HARMONY cohort for genetic profiling, which is currently the largest real-world comprehensive disease cohort of HAs in China. Notably, we imposed no restrictions on enrollment regarding AAO, family history, certain symptoms, or prior testing, nor did we limit screening to specific subtypes. The cohort encompasses 2096 probands recruited over a 17-year period mainly from the YRD region, and features systematic frameworks for genetic testing and molecular diagnostic management. The Yangtze River Delta, one of the most economically developed and densely populated areas in China, also exhibits a high burden of rare neurological diseases, along with great healthcare-seeking behavior and increased acceptance of genetic testing [41]. As major referral centers for rare neurological diseases at both regional and national levels, SAHZU and HSFU contribute this YRD-centered cohort, which is highly representative of the genetic characteristics of HAs encountered in the YRD and may also provide informative insights into the broader Chinese spectrum through its partial composition by out-of-region referrals.

**Fig. 5:**
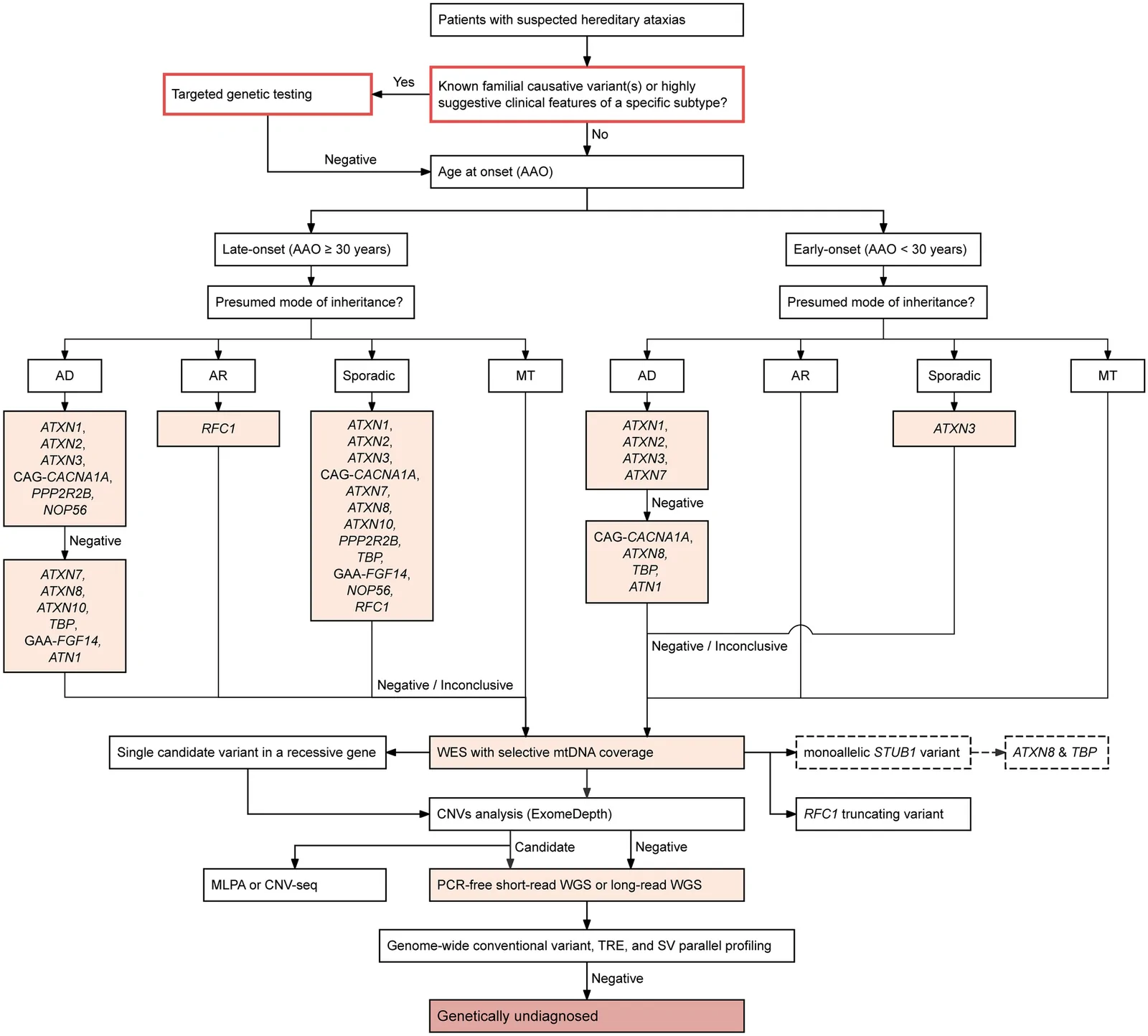
YRD-oriented candidate molecular diagnostic algorithm for hereditary ataxias. The algorithm adopts a stepwise design, prioritizing PCR-based screening for common TREs according to the AAO and presumed mode of inheritance, with rarer-TRE screening and NGS reserved for patients with negative or inconclusive initial results. Of note, pedigree- or phenotype-driven inspection for specific genes or variants (the steps outlined in red) remains the priority, before proceeding to broader genetic testing. The dashed boxes and arrow indicate results warranting clinical attention but with limited interpretive significance. AD: autosomal dominant; AR: autosomal recessive; CNV: copy number variant; MLPA: multiplex ligation-dependent probe amplification; MT: mitochondrial; SV: structural variant; TRE: tandem repeat expansion; WGS: whole genome sequencing; YRD: Yangtze River Delta

The genetic landscape of HAs in the HARMONY cohort is characterized by the predominance of a few common repeat-expansion subtypes, together with a long tail of diverse rare genotypes exhibiting remarkable genetic heterogeneity. Furthermore, the distribution remains uneven among the common subtypes themselves, with SCA3 represents the overwhelming majority. Such a skewed distribution supports an epidemiology-informed stepwise molecular diagnostic strategy that methodically prioritizes screening of common TREs before rarer-TRE and sequencing-based assays, enabling efficient and cost-effective detection across diverse potential pathogenic variant classes. Informed by the genetic profile of the HARMONY cohort, we further propose a YRD-oriented candidate molecular diagnostic algorithm as an operational implementation of this strategy to streamline the diagnostic process for clinical practice (Fig. 5).

The molecular diagnostic yield of 84.9% in the HARMONY cohort substantially exceeds those reported in other population-based cohorts globally, including a 36.7% yield in an Italian cohort [14] and a 65.3% yield in an Irish cohort [15]. The subtype distributions in these cohorts lack the predominance of SCA3, SCA2, and SCA1 seen in the HARMONY cohort, indicating that the differences in diagnostic rates are largely driven by population-specific genetic backgrounds. Within the Chinese context, our diagnostic yield was also higher than those reported in previous studies among patients with HAs from central China, where the diagnostic rates were 63.0% in Chen et al. [18] and 77.2% in Wan et al. [19]. We attribute the diagnostic advance to rigorous patient enrollment, expanded TRE loci screening, systematic molecular diagnostic classification, and comprehensive NGS analysis. To further benchmark the HARMONY cohort against these two Chinese cohorts, we performed compositional comparisons of genotype proportions. Given the methodological heterogeneity across these studies, data were aligned in three respects to enable valid pairwise comparisons: each comparison was restricted to the intersection of detection scopes within each study’s diagnostic workflow, cases without any candidate variants were excluded, and pathogenicity stratification for non-TRE variants was harmonized—HARMONY’s positive diagnoses were compared against Chen et al., and its genetic findings against Wan et al. Aligned to the autosomal dominant ataxia gene panel screened by Chen et al., HARMONY showed highly congruent genotype composition (*n* = 938 vs. 1,737; BCSI = 0.946; JSD = 0.017; Table S8), suggesting that the YRD dominant ataxia spectrum closely mirrors that of central China, albeit with relative enrichment of SCA12 and SCA36 in the HARMONY cohort. However, comparison with Wan et al., which included recessive ataxias, revealed appreciable divergence (*n* = 180 vs. 1804; BCSI = 0.821; JSD = 0.083; Table S9), largely driven by a lower SCA3 proportion and a higher fraction of non-TRE variants in Wan et al. Of note, the limited size of Wan et al. renders rare subtypes prone to disproportionate fluctuations under sampling variation, likely inflating the apparent divergence.

These within-China comparisons suggest that the overall genetic landscape is largely shared between the YRD and central China, with a few TRE subtypes predominating over a long tail of rare etiologies. This shared holistic profile supports the broader applicability in China of the stepwise strategy that prioritizes PCR-based analysis of common TREs and reserves rarer-TRE and sequencing-based assays for unresolved patients. An important consideration is that for the HARMONY cohort, where SCA1, 2, 3, 6, and 12 constitute 77.5% of HAs, classic PCR-based analysis remains a simple, economical, and practical method for resolving the majority of molecular diagnoses (with rare exceptions requiring interruption analysis in SCA1 and SCA2 for boundary alleles). Consequently, the epidemiology-informed stepwise strategy not only ensures high clinical utility but also effectively triages patients with potentially heterogeneous etiologies who genuinely require broader or advanced genetic assays. Still, the modest subtype-level differences observed across the Chinese cohorts indicate that converting this strategy into a practical diagnostic algorithm, particularly in terms of panel composition and testing order, requires further validation in multicenter cohorts with broader regional representation, during which the need for regional adjustment can be evaluated.

PCR-based methods, the standard approach for TRE characterization, struggle with the scalability required to address the heterogeneity of diverse TRE subtypes due to their locus-specific and labor-intensive nature; as such, the layout of TRE screening targets stands as the principal variable in tailoring the algorithm. Targeted NGS can interrogate conventional variants and CNVs in a high-throughput parallel manner. Although targeted-sequencing data can support candidate identification of selected TREs using dedicated bioinformatics tools, systematic TRE characterization remains constrained by capture-based chemistry, limited genomic scope, and inaccurate sizing [42, 43]. In this context, short-read WGS has emerged as a more comprehensive complement, enabling genome-wide parallel assessment of diverse variant classes, while improving candidate TRE identification through more uniform read-depth and repeat-associated sequence signals [43–45]. Nevertheless, short-read WGS is still primarily suited for flagging candidate TREs, typically followed by PCR-based confirmation, as accurate sizing and motif resolution of large expansions remain challenging [46]. The advent of long-read genomic or targeted sequencing directly addresses the difficulties in sequencing TREs, which enables precise quantification of repeat size and resolution of complex motifs, presenting another high-throughput method for molecular diagnosis [47]. Yet, for the few common TREs readily resolvable by PCR in Chinese HA patients, the high-throughput sequencing technologies represent an unwarranted escalation; their ideal role lies in collectively capturing the markedly heterogeneous long tail of rare subtypes. Therefore, we configured the rationalized molecular diagnostic strategy as a stepwise or sequential model and anticipate that it will prevail until the advanced sequencing technologies become substantially less costly or are proven to provide additional clinical value beyond basic TRE characterization. Such stepwise design is equally relevant in other global populations where the genetic landscape of HAs is similarly dominated by a few TRE subtypes tractable with PCR, especially in regions with limited economic development.

Rationalized molecular diagnostic strategies incorporating molecular epidemiological considerations are crucial for efficient management of HAs, aiding early molecular diagnosis and facilitating genetic counseling for patients and their families, helping reduce vertical transmission. Moreover, such strategies with the underlying genetic landscapes can accelerate the translation of genetic insights into precision medicine by not only enabling the rapid recruitment of genetically defined patients for clinical trials, but also by informing the strategic prioritization of HA subtypes for therapeutic development. The genetic landscapes can also serve as a real-world reference for developing locally adapted diagnostic tools. This is exemplified by the selection of TRE loci for targeted long-read sequencing (TLRS) panels. In the context of the HARMONY cohort, high-cost TLRS primarily serves as a crucial supplementary modality, deployed either after PCR excludes the common subtypes or to specifically characterize an individual challenging TRE. This deployment model suggests the need for customizable rather than fixed TLRS panels. Positioning TLRS as a sequential diagnostic modality is essential for facilitating its effective and pragmatic application in clinical practice.

Co-occurring variants in single probands were also recurrent in the HARMONY cohort. We identified co-occurring *STUB1* variants and *TBP*-i in eight patients and *STUB1* variants and *ATXN8* expansions in six patients. Among all these *STUB1* variants, 8 were private variants absent from gnomAD and the remaining six were ultra-rare variants with a minor allele frequency below 0.001%. In the absence of co-segregation data, the frequency of rare *STUB1* variants in patients with isolated *TBP*-i (8/12, 66.7%) and isolated *ATXN8* expansions (6/14, 42.9%) suggests the potential association between *STUB1* and *TBP,* and adds to the evidence for the hypothesis that *STUB1* variants may act as genetic modifiers in SCA8 as well. In addition, the recurrent co-occurrence of *TBP*-i and *ATXN8* expansions with variants in other HA genes alerts that these two genotypes still warrant broader genetic evaluation.

Moreover, based on the STR Panel C screening results from a subset of ‘Undiagnosed’ patients, we presume that SCA4, SCA31, SCA37, SCA51, and Friedreich’s ataxia are likely to be extremely rare in the Chinese population. This is consistent with previous studies, which similarly found absent frequencies of these subtypes [18, 48, 49]. Therefore, Panel C should remain a research-oriented TRE screening choice and is not recommended for routine clinical diagnostic use.

Despite the contributions, this study had certain limitations. While the retrospective design can underestimate the prevalence of co-occurrence across the cohort, cases with co-occurring TREs across the STR Panels A and B may also be missed, as the applied genetic testing workflow evaluates these panels in isolation. Additionally, this workflow bypasses Panel B screening for most patients with non-AD, early-onset ataxia, such that biallelic expansions at the TRE loci included in Panel B may remain undetected. It has been reported that in a pedigree with a non-AD inheritance pattern, biallelic GAA-*FGF14* expansions can precipitate an earlier onset of SCA27B [50]. Some cases uniformly classified as ‘Undiagnosed’ with DNA unavailable for subsequent genetic assessment may introduce some bias; yet retaining these cases with existing results still helps preserve the genetic landscape by maintaining a complete denominator. Moreover, the strict inclusion criteria resulted in the exclusion of patients with potential MSA-like sporadic ataxia, thereby underestimating the frequency of subtypes like SCA27B and CANVAS [51]. With detailed scales available only in a subset of patients, SDFS was used to maximize cohort-wide severity coverage; however, it is less granular and harmonized than the Scale for the Assessment and Rating of Ataxia and Friedreich Ataxia Rating Scale–Activities of Daily Living [52]. The limitations of PCR-based analysis call for further investigation to define the exact repeat counts and internal motifs of the long repeat regions in *ATXN10* and *NOP56*, which will require follow-up TLRS for these special subtypes.

## Conclusions

By establishing the HARMONY cohort, the largest comprehensive HA cohort in China to date (2096 probands), we delineated a genetic landscape characterized by the predominance of a few common repeat-expansion subtypes and a highly heterogeneous long tail of rare genotypes, achieving a molecular diagnostic yield of 84.9%. The resulting genetic profile informed a stepwise molecular diagnostic strategy that prioritizes screening of common TREs before broader or advanced genetic assays, with a YRD-oriented candidate algorithm proposed as its practical implementation to streamline the diagnostic process. This study provides an evidence base for early molecular diagnosis, subtype-prioritized therapeutic development, and the design of locally adapted diagnostic tools for HAs in China. Future validation in additional multicenter cohorts is warranted to assess the generalizability of the stepwise strategy and further calibrate its derived diagnostic algorithm.

## Supplementary Information

Additional file 1. **Text S1**. Routine clinical workup for excluding acquired ataxias. **Table S1**. Complete list of hereditary ataxia genes applied in this study. **Text S2**. Protocols for screening with STR Panels B and C. **Text S3**. WGS data generation and analysis pipeline. **Table S2**. Genetic features of the HA-like probands excluded after systematic genetic testing due to candidate variants in genes outside the curated scope of HAs.** Table S3**. Geographic distribution of probands by provincial-level administrative division and recruitment center. **Table S4**. Genotype frequency profiles across the HARMONY cohort, the YRD, the non-YRD, and the YRD provinces. **Table S5**. Pairwise similarity of genotype frequency profiles among the four YRD provinces. **Table S6**. Chi-square comparison of the distribution of genetic findings between the two medical centers. **Table S7**. Previously unreported genetic findings by our group attributed to conventional and structural variants identified by targeted NGS. **Figure S1**. Molecular characterization of the five probands identified with pathogenic CNVs. **Table** **S8**. Aligned genotype distribution of autosomal dominant ataxia between Chen et al. and the HARMONY cohort. **Table S9**. Aligned genotype distribution of ataxia between Wan et al. and the HARMONY cohort.

Additional file 2. Full uncropped gel image for Fig. S1b

## Data Availability

The data supporting the findings of this study are available from the corresponding author Zhi-Ying Wu upon reasonable request. The data are not publicly available due to privacy reasons.

## Abbreviations

AAO: Age at onset
AD: Autosomal dominant
AR: Autosomal recessive
BCSI: Bray–Curtis similarity index
CANVAS: Cerebellar ataxia, neuropathy and vestibular areflexia syndrome
CI: Confidence interval
CNV: Copy number variant
HAs: Hereditary ataxias
IQR: Inter-quartile range
JSD: Jensen–Shannon divergence
MLPA: Multiplex ligation-dependent probe amplification
MSA-C: Multiple system atrophy cerebellar type
mtDNA: Mitochondrial DNA
NGS: Next-generation sequencing
OR: Odds ratio
SCA: Spinocerebellar ataxia
SDFS: Spinocerebellar degeneration functional score
SRCA: Society for Research on the Cerebellum and Ataxias
STR: Short tandem repeat
TLRS: Targeted long-read sequencing
TRE: Tandem repeat expansion
WES: Whole exome sequencing
WGS: Whole genome sequencing
YRD: Yangtze River Delta
