# Supplementary material for: Large-scale genetic profiling of hereditary ataxias in China: implications for a stepwise molecular diagnostic strategy
Source: Transl Neurodegener. 2026 Sep 29;15:50. doi: 10.1186/s40035-026-00584-y (PMC13621623; doi:10.1186/s40035-026-00584-y)
Supplement: Supplementary file 1 [file 40035_2026_584_MOESM1_ESM.pdf]

Additional file 1: Supplementary material

Text S1. Routine clinical workup for excluding acquired ataxias.

To ensure the specific recruitment of hereditary ataxias (HAs), a routine clinical workup was implemented to exclude acquired etiologies (secondary causes). This exclusion process followed standard clinical practice guidelines appropriate for the study period and focused on identifying toxic, nutritional, immune-mediated, infectious, and structural causes of ataxia. The routine workup typically comprised:

1. Clinical History Review (Toxic & Environmental Exclusion)

A comprehensive review of medical history was conducted to exclude ataxias secondary to exogenous agents:

- **Chronic Intoxication:** History of chronic alcohol abuse meeting clinical criteria for alcoholic cerebellar degeneration.
- **Medication/Toxin Exposure:** Long-term use of ataxia-inducing medications (e.g., phenytoin, lithium, chemotherapy agents like cytarabine/5-fluorouracil) or exposure to heavy metals (e.g., mercury, lead).

2. Neuroimaging Assessment (Structural Exclusion)

Cranial magnetic resonance imaging (or computed tomography if unavailable) was the standard modality employed to rule out structural lesions directly explaining the cerebellar symptoms. Exclusion criteria included:

- **Vascular events:** Evidence of acute or chronic stroke (ischemic or hemorrhagic) involving the cerebellum or brainstem.
- **Space-occupying lesions:** Primary brain tumors, metastases, or abscesses.
- **Congenital/Structural malformations:** Arnold-Chiari malformation or Dandy-Walker variants.
- **Superficial siderosis:** Characteristic linear hypointensities along the pial surface suggestive of chronic subarachnoid bleeding.
- **Acquired demyelination:** Lesions suggestive of multiple sclerosis or other inflammatory demyelinating diseases.

3. Laboratory Screening Panel (Metabolic, Immune & Infectious Exclusion)

Based on clinical indication, selected laboratory tests were performed to evaluate potentially treatable or reversible causes:

- **Nutritional/Metabolic:** Vitamin B12, folate, and vitamin E levels, with further evaluation for acquired causes of vitamin E deficiency where indicated, and thyroid function tests.
- **Immune-mediated:**
  - **Autoimmune Cerebellar Ataxia:** Screening for anti-glutamic acid decarboxylase 65 antibodies in patients with subacute onset or co-existing autoimmune diabetes.
  - **Paraneoplastic Cerebellar Degeneration:** In cases with rapid progression (subacute onset), screening for a comprehensive panel of onconeuronal antibodies (e.g., anti-Yo, anti-Hu, anti-Ri, anti-Ma2, and anti-Tr) and tumor screening (computed tomography/positron emission tomography) was performed.
  - **Gluten Ataxia:** Screening for gluten sensitivity (anti-gliadin and anti-transglutaminase 6 antibodies) was considered in idiopathic sporadic cases where available.
- **Infectious:** Serological testing for syphilis (rapid plasma reagin/*Treponema pallidum* particle agglutination assay) and human immunodeficiency virus was performed to exclude infectious or opportunistic cerebellar involvement.

Table S1. Complete list of hereditary ataxia genes applied in this study.

Index	Gene	MDS Nomenclature	SRCA Classification
1	*ABCA2*	ATX-*ABCA2*	–
2	*ABCB7*	ATX-*ABCB7*	–
3	*ABHD12*	ATX-*ABHD12*	Primary (rare)
4	*ADCK3*/*COQ8A*	ATX-*ADCK3*	Primary
5	*ADPRHL2*/*ADPRS*	ATX-*ADPRHL2*	–
6	*AFG3L2*	ATX-*AFG3L2*	Primary (rare)
7	*AIFM1*	ATX-*AIFM1*	–
8	*ANO10*	ATX-*ANO10*	Primary
9	*APTX*	ATX-*APTX*	Primary
10	*ATCAY*	–	Primary (rare)
11	*ATM*	ATX-*ATM*	Primary
12	*ATN1*	ATX-*ATN1*	–
13	*MT-ATP6*	ATX-*MT-ATP6*	–
14	*ATXN1*	ATX-*ATXN1*	–
15	*ATXN10*	ATX-*ATXN10*	–
16	*ATXN2*	ATX-*ATXN2*	–
17	*ATXN3*	ATX-*ATXN3*	–
18	*ATXN7*	ATX-*ATXN7*	–
19	*ATXN8*/*ATXN8OS*	ATX-*ATXN8OS*	–
20	*BCKDHB*	ATX-*BCKDHB*	–
21	*BEAN1*	ATX-*BEAN1*	–
22	*BRAT1*	ATX-*BRAT1*	–
23	*CA8*	ATX-*CA8*	Primary (rare)
24	*CACNA1A*	ATX-*CACNA1A*	–
25	*CACNA1G*	ATX-*CACNA1G*	–
26	*CACNA2D2*	ATX-*CACNA2D2*	–
27	*CAPN1*	HSP/ATX-*CAPN1*	Primary (rare)
28	*CCDC88C*	ATX-*CCDC88C*	–
29	*CLCN2*	HSP/ATX-*CLCN2*	Primary (rare)
30	*COA7*	ATX-*COA7*	Primary (rare)
31	*COG5*	ATX-*COG5*	–
32	*COX20*	ATX-*COX20*	Primary (rare)
33	*CWF19L1*	ATX-*CWF19L1*	Primary (rare)
34	*CYP27A1*	ATX-*CYP27A1*	Primary
35	*CYP7B1*	HSP/ATX-*CYP7B1*	Primary (rare)
36	*DAB1*	ATX-*DAB1*	–
37	*DARS2*	ATX/HSP-*DARS2*	Primary (rare)
38	*DNAJC19*	ATX-*DNAJC19*	Primary (rare)
39	*DNMT1*	ATX-*DNMT1*	–
40	*DOCK3*	ATX-*DOCK3*	–
41	*EBF3*	ATX-*EBF3*	–
42	*ELOVL4*	ATX-*ELOVL4*	–
43	*ELOVL5*	ATX-*ELOVL5*	–

Table S1. Complete list of hereditary ataxia genes applied in this study. (continued)

Index	Gene	MDS Nomenclature	SRCA Classification
44	*FGF14*	ATX-*FGF14*	–
45	*FXN*	ATX-*FXN*	Primary
46	*GBA2*	HSP/ATX-*GBA2*	Primary (rare)
47	*GDAP2*	ATX-*GDAP2*	Primary (rare)
48	*GJC2*	ATX/HSP-*GJC2*	Primary (rare)
49	*GOSR2*	MYC/ATX-*GOSR2*	Primary (rare)
50	*GRID2*	ATX-*GRID2*	Primary (rare)
51	*GRM1*	–	Primary (rare)
52	*GRN*	ATX-*GRN*	Primary (rare)
53	*ITPR1*	ATX-*ITPR1*	Primary (rare)
54	*KCNA2*	ATX/HSP-*KCNA2*	–
55	*KCNC3*	ATX-*KCNC3*	–
56	*KCND3*	ATX-*KCND3*	–
57	*KCNJ10*	ATX-*KCNJ10*	Primary (rare)
58	*KIF1C*	HSP/ATX-*KIF1C*	Primary (rare)
59	*L2HGDH*	ATX-*L2HGDH*	Primary (rare)
60	*LMNB1*	ATX-*LMNB1*	–
61	*MRE11*	ATX-*MRE11A*	Primary (rare)
62	*MTPAP*	–	Primary (rare)
63	*NFASC*	ATX-*NFASC*	–
64	*NOP56*	ATX-*NOP56*	–
65	*NUS1*	ATX/MYC-*NUS1*	–
66	*OPHN1*	ATX-*OPHN1*	–
67	*OTC*	ATX-*OTC*	–
68	*PDYN*	ATX-*PDYN*	–
69	*PEX10*	ATX-*PEX10*	Primary (rare)
70	*PIBF1*	ATX-*PIBF1*	–
71	*PLP1*	HSP/ATX-*PLP1*	–
72	*PMPCA*	ATX-*PMPCA*	Primary (rare)
73	*PNKP*	ATX-*PNKP*	Primary (rare)
74	*PNPLA6*	ATX-*PNPLA6*	Primary (rare)
75	*POLG*	–	Primary
76	*POLR3A*	ATX/HSP-*POLR3A*	Primary (rare)
77	*POLR3B*	ATX-*POLR3B*	Primary (rare)
78	*PPP2R2B*	ATX-*PPP2R2B*	–
79	*PRKCG*	ATX-*PRKCG*	–
80	*PUM1*	ATX-*PUM1*	–
81	*RFC1*	ATX-*RFC1*	–
82	*RNF168*	ATX-*RNF168*	–
83	*RNF216*	ATX-*RNF216*	Primary (rare)
84	*RUBCN*	ATX-*RUBCN*	–
85	*SACS*	ATX/HSP-*SACS*	Primary
86	*SAMD9L*	ATX-*SAMD9L*	–
87	*SCYL1*	–	Primary (rare)

Table S1. Complete list of hereditary ataxia genes applied in this study. (continued)

Index	Gene	MDS Nomenclature	SRCA Classification
88	*SETX*	ATX-*SETX*	Primary
89	*SIL1*	ATX-*SIL1*	Primary
90	*SLC33A1*	ATX-*SLC33A1*	–
91	*SLC9A1*	–	Primary (rare)
92	*SNAP25*	ATX-*SNAP25b*	–
93	*SNX14*	ATX-*SNX14*	Primary (rare)
94	*SPG7*	HSP/ATX-*SPG7*	Primary
95	*SPTBN2*	ATX-*SPTBN2*	Primary (rare)
96	*SQSTM1*	ATX/DYT-*SQSTM1*	–
97	*STUB1*	ATX-*STUB1*	Primary (rare)
98	*SYNE1*	ATX-*SYNE1*	Primary
99	*TANGO2*	ATX-*TANGO2*	–
100	*TBC1D23*	ATX-*TBC1D23*	–
101	*TBP*	ATX-*TBP*	–
102	*TDP2*	–	Primary (rare)
103	*TGM6*	ATX-*TGM6*	–
104	*TMEM216*	ATX-*TMEM216*	–
105	*TMEM240*	ATX-*TMEM240*	–
106	*TPP1*	ATX/MYC-*TPP1*	Primary (rare)
107	*TSEN54*	ATX-*TSEN54*	–
108	*TTBK2*	ATX-*TTBK2*	–
109	*TTPA*	ATX-*TTPA*	Primary
110	*TUBB2A*	ATX-*TUBB2A*	–
111	*TWNK*	ATX-*C10orf2*	Primary
112	*UCHL1*	HSP/ATX-*UCHL1*	Primary (rare)
113	*VAMP1*	ATX/HSP-*VAMP1*	–
114	*VLDLR*	ATX-*VLDLR*	Primary (rare)
115	*VPS13D*	ATX/HSP-*VPS13D*	Primary (rare)
116	*WDR81*	ATX-*WDR81*	Primary (rare)
117	*XRCC1*	ATX-*XRCC1*	Primary (rare)
118	*THAP11*[a]	–	–
119	*ZFHX3*[a]	–	–

[a]*THAP11* and *ZFHX3* have recently been identified in association with HAs, but these two entities were not included in the complete list of HAs updated by the Genetic Nomenclature in Movement Disorders Study Group of the MDS in October 2022. We included both genes in our scope of HA research to reflect the latest scientific findings. Primary (rare) indicates primary autosomal recessive cerebellar ataxias classified as “rare ataxias or described only in few families” in the SRCA consensus statement.

MDS: Movement Disorder Society; SRCA: the Society for Research on the Cerebellum and Ataxias.

Text S2. Protocols for screening with STR Panels B and C.

Genomic DNA was amplified by PCR using KOD-FX polymerase (Toyobo, Osaka, Japan) in a 50 μL reaction volume. Each reaction mixture contained 25 μL of 2× PCR buffer for KOD-FX, 10 μL of 2 mM dNTPs (final concentration 0.4 mM), 1 μL of template DNA (50–200 ng), 3 μL of the primer mix (10 μM), and 1 μL (1.0 U) of KOD-FX polymerase. Nuclease-free water was added to bring the volume to 50 μL.

The thermal cycling protocol was performed on a ProFlex™ PCR System, 3 × 32-well (Applied Biosystems, Foster City, CA, USA) and consisted of an initial denaturation at 95°C for 5 min, followed by a touchdown PCR phase of seven cycles. Each touchdown cycle included denaturation at 95°C for 30 s, annealing for 30 s with a starting temperature of 65°C (decreasing by 1°C per cycle), and extension at 72°C for 2 min. Subsequently, 28 standard cycles were run, comprising denaturation at 95°C for 30 s, annealing at 58°C for 30 s, and extension at 72°C for 2 min. A final extension step was carried out at 72°C for 10 min.

Primers used for screening with STR Panels B and C are as follows:

Locus	Primer Name	Sequence	FL	RP
ATXN8	SCA8-F	FAM-5'-CCAATTCCTTGGCTAGACCCTGGGTC	√	√
	SCA8-R4	5'-CAAGAGAGAGCAACTAACTCAACA	√	/
	SCA8-P4	5'-TACGCATCCCAGTTTGAGACGCAGCAGCAGCAGCAG	/	√
	P3-1	5'-TACGCATCCCAGTTTGAGACG	/	√
ATXN10	SCA10-F	FAM-5'-AGATGGCAGAATGATAAACTCAATCA	√	√
	SCA10-R	5'-GCCTGGGCAACATAGAGAGACTT	√	/
	SCA10-P4	5'-TACGCATCCCAGTTTGAGACGAGAATAGAATAGAATAGAAT	/	√
	P3-1	5'-TACGCATCCCAGTTTGAGACG	/	√
NOP56	SCA36-F	FAM-5'-CATGGTGAGGAGTGGTTGCGGG	√	√
	SCA36-R	5'-TCAAACAGCACGTGCAACAGCAC	√	/
	SCA36-P4	5'-TACGCATCCCAGTTTGAGACGCAGGCCCAGGCC	/	√
	P3-1	5'-TACGCATCCCAGTTTGAGACG	/	√
FGF14	SCA27B-F	FAM-5'-CTTCTAGGCGCACTATATTGGGAA	√	√
	SCA27B-R	5'-CAGCAATCGTCAGTCAGTGTAAG	√	/
	SCA27B-R-FAM	FAM-5'-CAGCAATCGTCAGTCAGTGTAAG	/	√
	SCA27B-TTC-P4	5'-CAGGAAACAGCTATGACCTTCTTCTTCTTCTTCTTC	/	√
	SCA27B-TGCTTC-P4	5'-CAGGAAACAGCTATGACCTGCTTCTGCTTCTGCTTC	/	√
	SCA27B-GAA-P4	5'-CAGGAAACAGCTATGACCGAAGAAGAAGAAGAAGAA	/	√
	SCA27B-GAAGCA-P4	5'-CAGGAAACAGCTATGACCGAAGCAGAAGCAGAAGCA	/	√
	P3-2	5'-CAGGAAACAGCTATGACC	/	√
RFC1	RFC1-F	FAM-5'-CAAGTGATACTCCAGCTACAC	√	√
	RFC1-R	5'-GTGGGAGACAGGCCAATCACTT	√	/
	RFC1-AGGGG-P4	5'-CAGGAAACAGCTATGACCAGGGGAGGGGAGGGGAGGGGAGGGG	/	√
	RFC1-AAGGG-P4	5'-CAGGAAACAGCTATGACCAAGGGAAGGGAAGGGAAGGGAAGGG	/	√
	RFC1-AAAGG-P4	5'-CAGGAAACAGCTATGACCAAAGGAAAGGAAAGGAAAGGAAA	/	√
	RFC1-AAAAG-P4	5'-CAGGAAACAGCTATGACCAAAAGAAAAGAAAAGAAAAGAAAAG	/	√

(continued).

Locus	Primer Name	Sequence	FL	RP
RFC1	RFC1-AAAGGG-P4	5'-CAGGAAACAGCTATGACCAAAGGGAAAGGGAAAGGGAAAGGGAAAGGG	/	√
	RFC1-ACAGG-P4	5'-CAGGAAACAGCTATGACCACAGGACAGGACAGGACAGGACAGG	/	√
	P3-2	5'-CAGGAAACAGCTATGACC	/	√
THAP11	THAP11-F	FAM-5'-CAAAGGACGCTGAGTTGCGGCG	√	/
	THAP11-R	5'-AGCAGATACCAGGTTCGGCTG	√	/
ZFHX3	SCA4-F	FAM-5'-GCCAGTCTGTGGTGAACCTGCAA	√	/
	SCA4-R	5'-TTCTGTGTTTGTGCAAGGCCGA	√	/
BEAN1	SCA31-F	FAM-5'-ACTCCAACTGGGATGCAGTTTC	√	√
	SCA31-R	5'-CCAGATATCCCCAGTAATGCCTC	√	/
	SCA31-P4	5'-TACGCATCCCAGTTTGAGACGTTCCATTCCATTCCATTCCATTCCA	/	√
	P3-1	5'-TACGCATCCCAGTTTGAGACG	/	√
DAB1	SCA37-F	FAM-5'-GTCAGCCTCCCAGGTAAC	√	/
	SCA37-R	5'-CACACCTATAATCCCGACACTTTG	√	/
	SCA37-F2	FAM-5'-GAAGTGGTCCTCCCAAGTCA	/	√
	SCA37-TGAAA-P4	5'-CGTCTCAAACTGGGATGCGTAATGAAATGAAATGAAATGAAATGAA	/	√
	SCA37-TAAAA-P4	5'-CGTCTCAAACTGGGATGCGTAATAAAATAAAATAAAATAAAATAAA	/	√
	P3-3	5'-CGTCTCAAACTGGGATGCGTA	/	√
FXN	FXN-F1	FAM-5'-GATCACCTGAGGTCCGGAG	√	√
	FXN-R1	5'-GCGATCTTGGCTTAATG	√	/
	FXN-P4	5'-TACGCATCCCAGTTTGAGACGGAAGAAGAAGAAGAA	/	√
	P3-1	5'-TACGCATCCCAGTTTGAGACG	/	√

FL: flanking PCR; FAM: 6-carboxyfluorescein; RP: repeat-primed PCR; STR: short tandem repeat.

Text S3. WGS data generation and analysis pipeline.

For WGS, sample DNA was fragmented by Hieff Smearase (Yeasen, Shanghai, China) and was further selected to be between 200–500 bp by DNA clean beads. PCR-free library preparation was performed using the Hieff NGS DNA Library Prep Kit (Yeasen, Shanghai, China). Sequencing (150-bp paired-end reads) was performed on an Illumina NovaSeq 6000 platform, achieving an average coverage of approximately 35×. The sequencing data were processed using fastp to generate "clean reads" and aligned to the human reference genome (GRCh38) using the BWA aligner. Single nucleotide variants (SNVs) and small indels were jointly called across all samples using the GATK HaplotypeCaller version 4.0 and were filtered using the GATK Variant Quality Score Recalibration method with default parameters. Variant annotation was conducted using ANNOVAR. CNV re-calling from WGS data was performed using Manta (version 1.6.0), followed by annotation using AnnotSV.

CNV: copy number variant; NGS: next-generation sequencing; WGS: whole genome sequencing.

Table S2. Genetic features of the HA-like probands excluded after systematic genetic testing due to candidate variants in genes outside the curated scope of HAs.

No.	Gene	Zygosity	Variant	ACMG Classification	Disease
1	*NEU1*	HOM	NM_000434.4:c.544A>G:p.Ser182Gly	LP (PM2, PM3, PS3)	Sialidosis
2	*NEU1*	HOM	NM_000434.4:c.544A>G:p.Ser182Gly	LP (PM2, PM3, PS3)	Sialidosis
3	*NPC1*	CmpHET	NM_000271.5:c.2728G>A:p.Gly910Ser	P (PM1, PM2, PM3, PP2, PP3)	Niemann–Pick disease type C1
			NM_000271.5:c.2526T>A:p.Phe842Leu	P (PS1, PM1, PM2, PM5, PP2, PP3)	
4	*PRNP*	HET	NM_000311.5:c.305C>T:p.Pro102Leu	P (PS2, PS3, PM2, PM5, PP1, PP3)	Genetic prion disease
5	*C19orf12*	HET	NM_031448.6:c.300_304del:p.Glu100Aspfs*39	LP (PVS1, PM2)	Mitochondrial membrane protein-associated neurodegeneration
6	*DDHD2*	CmpHET	NM_001164232.2:c.-8-13_-8-9del	VUS (PM2)	Hereditary spastic paraplegia 54
			NM_001164232.2:c.622G>A:p.Gly208Arg	VUS (PM2, PP3)	
7	*MFSD8*	CmpHET	NM_152778.3:c.863+1G>A	P (PVS1, PM2, PM3)	Neuronal ceroid lipofuscinosis 7
			NM_152778.3:c.1268C>G:p.Ala423Gly	VUS (PM2, PM3, PP2)	

ACMG classification includes pathogenic (P), likely pathogenic (LP), and variant of uncertain significance (VUS), with supporting evidence listed in parentheses according to the ACMG standards and guidelines.

ACMG: the American College of Medical Genetics and Genomics; CmpHET: compound heterozygous; HET: heterozygous; HOM: homozygous.

Table S3. Geographic distribution of probands by provincial-level administrative division and recruitment center.

Rank	Provincial-Level Administrative Divisions	Total	SAHZU	HSFU
1	Zhejiang	851	636	215
2	Jiangsu	315	30	285
3	Anhui	244	84	160
4	Shanghai	160	2	158
5	Jiangxi	110	55	55
6	Henan	60	34	26
7	Fujian	59	22	37
8	Hubei	54	19	35
9	Shandong	34	10	24
10	Sichuan	32	15	17
11	Hunan	27	14	13
12	Heilongjiang	18	10	8
13	Yunnan	13	9	4
14	Guizhou	12	10	2
15	Liaoning	10	5	5
16	Chongqing	10	4	6
17	Guangdong	9	5	4
18	Shaanxi	9	6	3
19	Hebei	8	4	4
20	Inner Mongolia	7	5	2
21	Xinjiang	7	3	4
22	Jilin	5	2	3
23	Gansu	4	2	2
24	Tianjin	2	0	2
25	Shanxi	2	1	1
26	Guangxi	2	1	1
27	Qinghai	2	1	1
28	Ningxia	1	1	0
29	Hainan	1	1	0
**Total**		2068	991	1077

Geographic information was unavailable for 28 of the 2096 probands (SAHZU, n = 6; HSFU, n = 22).

HSFU: Huashan Hospital, Fudan University; SAHZU: the Second Affiliated Hospital of Zhejiang University School of Medicine.

Table S4. Genotype frequency profiles across the HARMONY cohort, the YRD, the non-YRD, and the YRD provinces.

Genotypes	HARMONY Cohort (n = 2096)	YRD (n = 1570)	non-YRD (n = 498)	Zhejiang (n = 851)	Jiangsu (n = 315)	Anhui (n = 244)	Shanghai (n = 160)
ATXN3	1249 (59.6%)	964 (61.4%)	274 (55.0%)	507 (59.6%)	208 (66.0%)	146 (59.8%)	103 (64.4%)
ATXN2	150 (7.2%)	110 (7.0%)	39 (7.8%)	52 (6.1%)	19 (6.0%)	30 (12.3%)	9 (5.6%)
ATXN1	139 (6.6%)	98 (6.2%)	40 (8.0%)	40 (4.7%)	26 (8.3%)	20 (8.2%)	12 (7.5%)
CAG-*CACNA1A*	51 (2.4%)	31 (2.0%)	20 (4.0%)	26 (3.1%)	2 (0.6%)	0	3 (1.9%)
NOP56	36 (1.7%)	21 (1.3%)	15 (3.0%)	12 (1.4%)	3 (1.0%)	3 (1.2%)	3 (1.9%)
PPP2R2B	35 (1.7%)	29 (1.8%)	4 (0.8%)	11 (1.3%)	8 (2.5%)	0	10 (6.2%)
ATXN7	20 (1.0%)	16 (1.0%)	4 (0.8%)	9 (1.1%)	4 (1.3%)	2 (0.8%)	1 (0.6%)
SACS	17 (0.8%)	14 (0.9%)	3 (0.6%)	10 (1.2%)	1 (0.3%)	2 (0.8%)	1 (0.6%)
SETX	14 (0.7%)	8 (0.5%)	6 (1.2%)	4 (0.5%)	1 (0.3%)	3 (1.2%)	0
SYNE1	11 (0.5%)	8 (0.5%)	3 (0.6%)	7 (0.8%)	1 (0.3%)	0	0
ATXN8	9 (0.4%)	8 (0.5%)	1 (0.2%)	4 (0.5%)	2 (0.6%)	0	2 (1.2%)
CACNA1A	9 (0.4%)	5 (0.3%)	4 (0.8%)	3 (0.4%)	0	2 (0.8%)	0
COX20	9 (0.4%)	8 (0.5%)	1 (0.2%)	8 (0.9%)	0	0	0
ATN1	8 (0.4%)	8 (0.5%)	0	2 (0.2%)	3 (1.0%)	2 (0.8%)	1 (0.6%)
STUB1;*TBP-i*	8 (0.4%)	6 (0.4%)	2 (0.4%)	2 (0.2%)	2 (0.6%)	2 (0.8%)	0
GAA-*FGF14*	7 (0.3%)	5 (0.3%)	2 (0.4%)	4 (0.5%)	0	0	1 (0.6%)
RFC1	6 (0.3%)	6 (0.4%)	0	6 (0.7%)	0	0	0
STUB1;*ATXN8*	6 (0.3%)	5 (0.3%)	1 (0.2%)	3 (0.4%)	1 (0.3%)	1 (0.4%)	0
TBP	6 (0.3%)	5 (0.3%)	1 (0.2%)	3 (0.4%)	1 (0.3%)	0	1 (0.6%)
mtDNA	6 (0.3%)	5 (0.3%)	0	5 (0.6%)	0	0	0
ATXN3;*ATXN2*	5 (0.2%)	3 (0.2%)	2 (0.4%)	1 (0.1%)	1 (0.3%)	1 (0.4%)	0
SPG7	5 (0.2%)	4 (0.3%)	1 (0.2%)	3 (0.4%)	0	1 (0.4%)	0
STUB1	5 (0.2%)	4 (0.3%)	1 (0.2%)	2 (0.2%)	1 (0.3%)	1 (0.4%)	0
TBP-i	5 (0.2%)	2 (0.1%)	3 (0.6%)	1 (0.1%)	1 (0.3%)	0	0
ADCK3	4 (0.2%)	0	1 (0.2%)	0	0	0	0
ATM	4 (0.2%)	2 (0.1%)	2 (0.4%)	1 (0.1%)	0	1 (0.4%)	0
PRKCG	4 (0.2%)	3 (0.2%)	1 (0.2%)	2 (0.2%)	0	1 (0.4%)	0
AFG3L2	3 (0.1%)	3 (0.2%)	0	3 (0.4%)	0	0	0
KCNC3	3 (0.1%)	1 (0.1%)	2 (0.4%)	1 (0.1%)	0	0	0
ATXN10	2 (0.1%)	0	2 (0.4%)	0	0	0	0
ATXN2;*TBP*-i	2 (0.1%)	1 (0.1%)	1 (0.2%)	1 (0.1%)	0	0	0
ITPR1	2 (0.1%)	1 (0.1%)	1 (0.2%)	0	0	0	1 (0.6%)
KCND3	2 (0.1%)	1 (0.1%)	1 (0.2%)	0	0	1 (0.4%)	0
NOP56;*TBP*-i	2 (0.1%)	2 (0.1%)	0	2 (0.2%)	0	0	0
SPTBN2	2 (0.1%)	1 (0.1%)	0	1 (0.1%)	0	0	0
TMEM240	2 (0.1%)	2 (0.1%)	0	0	1 (0.3%)	1 (0.4%)	0
TTPA	2 (0.1%)	1 (0.1%)	1 (0.2%)	0	1 (0.3%)	0	0
ADPRHL2	1 (0.0%)	1 (0.1%)	0	1 (0.1%)	0	0	0
ANO10	1 (0.0%)	1 (0.1%)	0	1 (0.1%)	0	0	0
APTX	1 (0.0%)	0	0	0	0	0	0
ATXN1;*TBP*-i	1 (0.0%)	0	1 (0.2%)	0	0	0	0
ATXN3;*PPP2R2B*	1 (0.0%)	1 (0.1%)	0	1 (0.1%)	0	0	0

Table S4. Genotype frequency profiles across the HARMONY cohort, the YRD, the non-YRD, and the YRD provinces. (continued)

Genotypes	HARMONY Cohort (n = 2096)	YRD (n = 1570)	non-YRD (n = 498)	Zhejiang (n = 851)	Jiangsu (n = 315)	Anhui (n = 244)	Shanghai (n = 160)
ATXN3;TBP-i	1 (0.0%)	1 (0.1%)	0	1 (0.1%)	0	0	0
CACNA1G	1 (0.0%)	0	0	0	0	0	0
CWF19L1	1 (0.0%)	1 (0.1%)	0	1 (0.1%)	0	0	0
GDAP2	1 (0.0%)	1 (0.1%)	0	1 (0.1%)	0	0	0
KIF1C	1 (0.0%)	0	1 (0.2%)	0	0	0	0
MRE11	1 (0.0%)	1 (0.1%)	0	0	1 (0.3%)	0	0
NOP56;*ATXN8*	1 (0.0%)	1 (0.1%)	0	1 (0.1%)	0	0	0
PMPCA	1 (0.0%)	1 (0.1%)	0	0	0	1 (0.4%)	0
POLG	1 (0.0%)	1 (0.1%)	0	1 (0.1%)	0	0	0
POLR3A	1 (0.0%)	1 (0.1%)	0	1 (0.1%)	0	0	0
RFC1-E/T	1 (0.0%)	0	1 (0.2%)	0	0	0	0
VPS13D	1 (0.0%)	1 (0.1%)	0	1 (0.1%)	0	0	0
No Findings	229 (10.9%)	167 (10.6%)	56 (11.2%)	105 (12.3%)	27 (8.6%)	23 (9.4%)	12 (7.5%)

HARMONY: Hereditary Ataxia Research on Multi-Omics and Neuroclinical Insights in the Yangtze Delta; YRD: Yangtze River Delta.

Table S5. Pairwise similarity of genotype frequency profiles among the four YRD provinces.

Rank	Comparison	Bray–Curtis Similarity Index	Jensen–Shannon Divergence
1	Jiangsu vs. Shanghai	0.916	0.036
2	Jiangsu vs. Anhui	0.878	0.054
3	Zhejiang vs. Jiangsu	0.858	0.056
4	Zhejiang vs. Anhui	0.854	0.080
5	Zhejiang vs. Shanghai	0.848	0.067
6	Anhui vs. Shanghai	0.836	0.100

Table S6. Chi-square comparison of the distribution of genetic findings between the two medical centers.

Categories	SAHZU	HSFU	Residuals (SAHZU/HSFU)	Difference (percentage points)	P	P_{BH}
ATXN3	526 (52.8%)	723 (65.8%)	-2.79/2.66	13.03	**0.005**	**0.021**
Others	135 (13.5%)	72 (6.6%)	3.68/-3.51	6.99	**<0.001**	**0.002**
No Findings	134 (13.4%)	95 (8.6%)	2.40/-2.29	4.80	**0.016**	**0.043**
CAG-*CACNA1A*	31 (3.1%)	20 (1.8%)	1.37/-1.30	1.29	0.17	0.34
PPP2R2B	12 (1.2%)	23 (2.1%)	-1.14/1.09	0.89	0.25	0.41
NOP56	21 (2.1%)	15 (1.4%)	0.94/-0.89	0.74	0.35	0.46
ATXN2	73 (7.3%)	77 (7.0%)	0.20/-0.19	0.32	0.84	0.89
ATXN1	65 (6.5%)	74 (6.7%)	-0.14/0.13	0.21	0.89	0.89
**Total**	997 (100.0%)	1099 (100.0%)	–	–	**<0.001**	–

P_{BH}: P value adjusted for multiple comparisons using the Benjamini–Hochberg method.

Table S7. Previously unreported genetic findings by our group attributed to conventional and structural variants identified by targeted NGS.

Patient ID	Sex	AAO (years)	Suspected Inheritance	Gene	Zygosity	Family Segregation	Variant	gnomAD (aggregated)	In Silico Predictions (REVEL/AlphaMissense /VARITY/PrimateAI/Splice AI)	Other Findings	HGMD	ClinVar	ACMG Classification	Molecular Diagnosis
HS_00034	F	6	Sporadic	*TTPA*	CmpHET	TRUE	NM_000370.3:c.489G>A:p.Trp163*	NA	NA/NA/NA/NA/B	.	Novel	Novel	LP (PVS1, PM2)	*Definitive*
						TRUE	NM_000370.3:c.224del:p.Lys75Serfs*9	NA	NA/NA/NA/NA/B		Novel	Novel	LP (PVS1, PM2)	
HS_00050	F	4	Sporadic	*TMEM240*	HET	NA	NM_001114748.1:c.509C>T:p.Pro170Leu	NA	U/U/D/D/B	.	Known	P	LP (PS4, PM2, PP1)	*Definitive*
HS_00051	F	29	Sporadic	*STUB1*	HET	TRUE	NM_005861.4:c.728C>T:p.Pro243Leu	4E-06	U/D/D/D/B	*TBP* (36/43)	Known	Conflict	LP (PS4, PM1, PM2, PP2)	*Unclear*
HS_00054	F	29	Sporadic	*STUB1*	HET	de novo	NM_005861.4:c.865_867delinsATA:p.Val289Ile	NA	NA/NA/NA/NA/NA	*TBP* (36/45)	Novel	Novel	LP (PS2, PM2, PP2)	*Unclear*
HS_00067	M	35	AR	*SYNE1*	HOM	NA	NM_033071.4:c.13207C>T:p.Arg4403*	1.2E-05	NA/NA/NA/NA/NA	.	Known	LP	LP (PVS1, PS4, PM2)	*Definitive*
HS_00096	F	42	AR	*SPG7*	HOM	NA	NM_003119.4:c.2162A>T:p.Asn721Ile	NA	D/U/D/U/B	.	Novel	Novel	LP (PM2, PM5, PP3)	*Definitive*
HS_00111	F	33	Sporadic	*PRKCG*	HET	NA	NM_002739.5:c.331C>A:p.Pro111Thr	NA	D/D/D/D/B	.	Novel	Novel	LP (PM1, PM2, PP2, PP3)	*Definitive*
HS_00195	F	33	AD	*STUB1*	HET	NA	NM_005861.4:c.761G>A:p.Arg254His	3.2E-06	D/D/D/D/B	*ATXN8* (17/99)	Novel	Novel	LP (PM1, PM2, PM5, PP2, PP3)	*Unclear*
HS_00197	M	23	Sporadic	*STUB1*	HET	NA	NM_005861.4:c.154G>A:p.Ala52Thr	NA	D/D/D/D/B	*ATXN8* (25/116)	Novel	VUS	VUS (PM2, PP2, PP3)	*Unclear*
HS_00208	M	20	Sporadic	*KCND3*	HET	NA	NM_001378969.1:c.1070C>T:p.Ser357Leu	NA	D/D/D/D/B	.	Known	LP	P (PS4, PM1, PM2, PP2, PP3)	*Definitive*
HS_00237	M	15	Sporadic	*SACS*	CmpHET	TRUE	NM_014363.6:c.171+2T>A	NA	NA/NA/NA/NA/S	.	Novel	Novel	LP (PVS1, PM2)	*Unclear*
						TRUE	NM_014363.6:c.1325G>A:p.Gly442Glu	4E-06	U/D/D/U/B		Novel	Novel	VUS (PM2, PM3)	
HS_00250	F	35	Sporadic	*STUB1*	HET	TRUE	NM_005861.4:c.728C>T:p.Pro243Leu	4E-06	U/D/D/D/B	*TBP* (34/43)	Novel	Conflict	LP (PS4, PM1, PM2, PP2)	*Unclear*
HS_00274	F	15	AD	*KCNC3*	HET	TRUE	NM_004977.3:c.1259G>A:p.Arg420His	NA	D/D/D/D/B	.	Known	P	P (PS3, PS4, PM1, PM2, PP1, PP3)	*Definitive*
HS_00340	M	23	Sporadic	*SETX*	CmpHET	TRUE	NM_015046.7:c.3327dup:p.Cys1110Metfs*20	NA	NA/NA/NA/NA/B	.	Novel	Novel	LP (PVS1, PM2)	*Definitive*
						de novo	NM_015046.7:c.6461C>T:p.Thr2154Met	4E-06	D/D/D/U/B	.	Novel	Novel	LP (PM2, PS2, PP3)	
HS_00350	M	19	Sporadic	*CACNA1A*	HET	NA	NM_001127222.2:c.889G>A:p.Gly297Arg	NA	D/D/D/D/B	.	Known	P	P (PS2, PM1, PM2, PP2, PP3)	*Definitive*
HS_00419	M	7	AR	*SACS*	HOM	NA	NM_014363.6:c.9647_9658delinsGAATTG: p.Asp3216_Ser3220delinsGlyIleAla	NA	NA/NA/NA/NA/NA	.	Novel	Novel	VUS (PM2, PM4)	*Probable*
HS_00423	F	19	AD	*STUB1*	HET	NA	NM_005861.4:c.146A>G:p.Tyr49Cys	NA	D/D/D/D/B	*ATXN8* (17/128)	Known	Conflict	VUS (PM2, PP2, PP3)	*Unclear*
HS_00465	F	20	Sporadic	*APTX*	CmpHET	TRUE	NM_001195248.2:c.739C>T:p.Arg247*	1.4E-05	NA/NA/NA/NA/M	.	Known	P	P (PVS1, PM2, PM3)	*Definitive*
						TRUE	NM_001195248.2:c.617C>T:p.Pro206Leu	2.8E-05	D/D/D/U/B		Known	P	LP (PM2, PM3, PP3)	
HS_00474	F	8	Sporadic	*ATM*	CmpHET	TRUE	NM_000051.4:c.6219_6220del:p.Cys2074Profs*13	NA	NA/NA/NA/NA/B	.	Novel	Novel	P (PVS1, PM2, PM5)	*Definitive*
						TRUE	NM_000051.4:c.6573-1G>A	NA	NA/NA/NA/NA/S		Novel	LP	LP (PVS1, PM2)	
HS_00498	M	NA	NA	*SACS*	CmpHET	TRUE	NM_014363.6:c.10466_10467del:p.Ser3489Leufs*2	NA	NA/NA/NA/NA/B	.	Known	P	P (PVS1, PM2, PM3)	*Definitive*
						TRUE	NM_014363.6:c.5719C>T:p.Arg1907*	4E-06	NA/NA/NA/NA/B		Known	P	P (PVS1, PM2, PM3)	
HS_00499	F	4	AD	*CACNA1A*	HET	NA	NM_001127222.2:c.2764dup:p.Arg922Profs*145	NA	NA/NA/NA/NA/B	.	Novel	Novel	LP (PVS1, PM2)	*Definitive*
HS_00500	F	5	AD	*CACNA1A*	HET	NA	NM_001127221.2:c.5569C>T:p.Arg1857*	NA	NA/NA/NA/NA/B	.	Known	P	P (PVS1, PS4, PM2, PP1)	*Definitive*

Table S7. Previously unreported genetic findings by our group attributed to conventional and structural variants identified by targeted NGS. (continued)

Patient ID	Sex	AAO (years)	Suspected Inheritance	Gene	Zygosity	Family Segregation	Variant	gnomAD (aggregated)	In Silico Predictions (REVEL/AlphaMissense /VARITY/PrimateAI/Splice AI)	Other Findings	HGMD	ClinVar	ACMG Classification	Molecular Diagnosis
HS_00504	F	NA	AD	*CACNA1G*	HET	NA	NM_018896.5:c.5144G>A:p.Arg1715His	NA	D/D/D/D/B	.	Known	P	P (PS3, PS4, PM2, PP1, PP2, PP3)	*Definitive*
HS_04566	M	54	AD	*CACNA1A*	HET	NA	NM_001127222.2:c.3613G>T:p.Glu1205*	NA	NA/NA/NA/NA/B	.	Novel	Novel	LP (PVS1, PM2)	*Definitive*
HS_05350	F	10	AD	*ITPR1*	HET	NA	NM_001378452.1:c.4123A>G:p.Met1375Val	NA	U/NA/D/U/B	.	Novel	Novel	VUS (PM2, PP2)	*Unclear*
HS_06738	F	21	AR (consanguineous)	*SETX*	HOM	NA	NM_015046.7:c.498+6T>G	NA	NA/NA/NA/NA/S	.	Novel	Novel	VUS (PM2, PP3)	*Probable*
HS_12498	F	39	Sporadic	*RFC1*	HET	NA	NM_002913.5:c.1157T>G:p.Leu386*	NA	NA/NA/NA/NA/B	*RFC1* [(AAAAG)$_{11}$/(AAGGG)$_{exp}$]	Novel	Novel	LP (PVS1, PM2)	*Unclear*
SZ_13886	F	39	AR	*STUB1*	HET	NA	NM_005861.4:c.101A>G:p.Asn34Ser	NA	U/D/D/D/B	*TBP* (38/45)	Known	VUS	VUS (PM2, PP2)	*Unclear*
SZ_14512	M	19	Sporadic	*STUB1*	HET	NA	NM_005861.4:c.278C>T:p.Ser93Phe	NA	U/D/D/D/B	.	Novel	Novel	VUS (PM2, PP2)	*Unclear*
SZ_15026	M	43	AR	*AFG3L2*	HET	NA	NM_006796.3:c.2059A>G:p.Lys687Glu	NA	D/D/D/D/B	.	Known	Novel	LP (PM1, PM2, PP2, PP3)	*Definitive*
SZ_15119	M	36	Sporadic	*SYNE1*	HET	NA	NM_182961.4:c.25189del:p.Glu8397Lysfs*21	0	NA/NA/NA/NA/NA	.	Novel	Novel	LP (PVS1, PM2)	*Unclear*
					HET	NA	NM_182961.4:c.1941dup:p.Arg648Serfs*22	1.5E-05	NA/NA/NA/NA/NA		Known	P	P (PVS1, PS4, PM2)	
SZ_15880	F	33	AD	*STUB1*	HET	NA	NM_005861.4:c.134C>G:p.Ala45Gly	NA	D/D/D/D/B	*TBP* (36/45)	Novel	Novel	VUS (PM2, PP2, PP3)	*Unclear*
SZ_17101	M	27	Sporadic	*AFG3L2*	HET	NA	NM_006796.3:c.2393A>G:p.*798Trpext*32	NA	NA/NA/NA/NA/B	.	Novel	Novel	VUS (PM2, PM4)	*Probable*
SZ_17427	F	12	AD	*KCNC3*	HET	NA	NM_004977.3:c.1268G>A:p.Arg423His	0	D/D/D/D/B	.	Known	P	P (PS2, PS3, PM1, PM2, PM5, PP1, PP3)	*Definitive*
SZ_20095	M	42	AR	*STUB1*	HET	NA	NM_005861.4:c.194A>C:p.Asn65Thr	NA	D/D/D/D/B	.	Novel	Novel	LP (PM1, PM2, PM5, PP2, PP3)	*Unclear*
SZ_20148	F	26	Sporadic	*POLR3A*	CmpHET	TRUE	NM_007055.4:c.1771-7C>G	1.2E-04	NA/NA/NA/NA/B	.	Known	Conflict	P (PS3, PM2, PM3)	*Probable*
						TRUE	NM_007055.4:c.3892G>T:p.Gly1298Cys	NA	D/D/D/D/B		Novel	Novel	VUS (PM2, PP2, PP3)	
SZ_21502	F	28	Sporadic	*CACNA1A*	HET	de novo	NM_001127222.2:c.6745C>A:p.Pro2249Thr	0	D/D/D/D/B	.	Novel	Novel	VUS (PM2, PP2, PP3)	*Probable*
SZ_21959	M	37	AR (consanguineous)	*SPG7*	HOM	NA	NM_003119.4:c.1846delC:p.Leu616Serfs*36	NA	NA/NA/NA/NA/B	.	Novel	Novel	LP (PVS1, PM2)	*Definitive*
SZ_22015	M	36	AD	*PRKCG*	HET	TRUE	NM_002739.4:c.301C>T:p.His101Tyr	0	D/D/D/D/B	.	Known	P	P (PS4, PM1, PM2, PM5, PP2, PP3)	*Definitive*
SZ_22247	F	32	AR	*STUB1*	HET	TRUE	NM_005861.4:c.478G>A:p.Glu160Lys	8E-06	B/B/D/D/B	*TBP* (36/43)	Novel	Novel	VUS (PM1, PM2, PP2)	*Unclear*
SZ_22863	M	33	Sporadic	*STUB1*	HET	TRUE	NM_005861.4:c.739C>T:p.Pro247Ser	NA	U/D/D/D/B	*TBP* (36/42)	Novel	Novel	VUS (PM1, PM2, PP2)	*Unclear*
SZ_23396	F	43	Sporadic	*STUB1*	HET	NA	NM_005861.4:c.721C>T:p.Arg241Trp	8E-06	U/U/D/D/B	*ATXN8* (38/105)	Known	LP	P (PS4, PM1, PM2, PM5, PP2)	*Unclear*
SZ_23986	M	49	AR	*SPG7*	CmpHET	NA	NM_003119.4:c.637C>T:p.Arg213*	4.3E-05	NA/NA/NA/NA/U	.	Known	P	P (PVS1, PM2, PS4)	*Probable*
						NA	NC_000016.10:g.89532845_89549420del	NA	NA/NA/NA/NA/NA		Novel	Novel	VUS (2E_0.45)	
SZ_24348	F	35	AR	*SPG7*	HOM	NA	NM_003119.4:c.861+1G>C	8E-06	NA/NA/NA/NA/S	.	Novel	P	P (PVS1, PM2, PS4)	*Definitive*
SZ_24521	M	50	AR	*STUB1*	HET	NA	NM_005861.4:c.101A>G:p.Asn34Ser	NA	U/D/D/D/B	*ATXN8* (18/104)	Known	VUS	VUS (PM2, PP2)	*Unclear*
SZ_25184	M	20	Sporadic	*KCND3*	HET	NA	NM_001378969.1:c.883T>G:p.Phe295Val	NA	D/D/D/D/B	.	Novel	Novel	LP (PM1, PM2, PP2, PP3)	*Definitive*

Table S7. Previously unreported genetic findings by our group attributed to conventional and structural variants identified by targeted NGS. (continued)

Patient ID	Sex	AAO (years)	Suspected Inheritance	Gene	Zygosity	Family Segregation	Variant	gnomAD (aggregated)	In Silico Predictions (REVEL/AlphaMissense /VARITY/PrimateAI/Splice AI)	Other Findings	HGMD	ClinVar	ACMG Classification	Molecular Diagnosis
SZ_25450	F	13	Sporadic	*CACNA1A*	HET	de novo	NM_001127222.2:c.1994C>T:p.Thr665Met	NA	D/D/D/D/B	.	Known	P	P (PS3, PS4, PM2, PP1, PP2, PP3)	*Definitive*
SZ_25595	F	7	Sporadic	*TMEM240*	HET	de novo	NM_001114748.2:c.196G>A:p.Gly66Arg	NA	U/D/D/D/S	.	Known	Conflict	LP (PS2, PM2, PM6)	*Definitive*
SZ_25945	M	16	AR	*SETX*	CmpHET	TRUE	NM_015046.7:c.6695G>A:p.Arg2232His	1E-06	D/D/D/B/B	.	Novel	VUS	LP (PM2, PM5, PP3)	*Definitive*
						TRUE	NC_000009.12:g.132325334_132350327del	NA	NA/NA/NA/NA/NA		Novel	Novel	LP (2C_0.90)	
SZ_26646	F	37	Sporadic	*STUB1*	HET	NA	NM_005861.4:c.760C>T:p.Arg254Cys	8E-06	U/D/D/D/U	*TBP* (35/42)	Known	VUS	LP (PM1, PM2, PM5, PP2)	*Unclear*
SZ_27460	F	5	Sporadic	*SACS*	HOM	NA	NM_014363.6:c.4756_4760del:p.Asn1586Tyrfs*3	1.3E-05	NA/NA/NA/NA/NA	.	Known	P	P (PVS1, PM2, PM3)	*Definitive*
SZ_27476	M	17	AR	*SETX*	HOM	NA	NC_000009.12:g.132331125_132341167del	NA	NA/NA/NA/NA/NA	.	Novel	Novel	LP (2E_0.90)	*Definitive*
SZ_27517	M	51	AR	*MT-ATP6*	.	TRUE	NC_012920.1:m.9174T>TC	0	NA/NA/NA/NA/NA	.	.	Novel	VUS (PVS1, PM2)	*Probable*
SZ_30306	F	41	Sporadic	*SACS*	CmpHET	TRUE	NM_014363.6:c.4933C>T:p.Arg1645*	6E-06	NA/NA/NA/NA/B	.	Known	P	P (PVS1, PM2, PM3)	*Unclear*
						TRUE	NM_014363.6:c.1076A>G:p.Tyr359Cys	1E-06	U/U/B/U/B		Novel	Novel	VUS (PM2, PM3)	
SZ_30914	F	1	Sporadic	*ITPR1*	HET	de novo	NM_001378452.1:c.805C>T:p.Arg269Trp	0	D/D/D/D/B	.	Known	P	P (PM1, PM2, PM5, PP1, PP2, PP3, PS2, PS3)	*Definitive*
SZ_31007	F	46	Sporadic	*AFG3L2*	HET	NA	NM_006796.3:c.2068A>G:p.Ser690Gly	NA	D/D/D/U/B	.	Novel	Novel	LP (PM1, PM2, PP2, PP3)	*Definitive*
SZ_31110	F	26	AR (consanguineous)	*SYNE1*	HOM	NA	NM_182961.4:c.6622G>T:p.Glu2208*	NA	NA/NA/NA/NA/NA	.	Novel	Novel	LP (PVS1, PM2)	*Definitive*
SZ_31189	M	45	Sporadic	*SACS*	HET	TRUE	NM_014363.6:c.11466del:p.Lys3822Asnfs*9	0	NA/NA/NA/NA/B	.	Novel	Novel	LP (PVS1, PM2)	*Definitive*
					HET	TRUE	NM_014363.6:c.5732dup:p.Phe1912Valfs*23	0	NA/NA/NA/NA/B		Novel	Novel	LP (PVS1, PM2)	
SZ_31306	M	35	Sporadic	*SPG7*	CmpHET	TRUE	NM_003119.4:c.2246del:p.Pro749Argfs*9	0	NA/NA/NA/NA/B	.	Novel	Novel	LP (PVS1, PM2)	*Definitive*
						TRUE	NC_000016.10:g.89505632_89538006del	NA	NA/NA/NA/NA/NA		Novel	Novel	P (2C_0.9, 4L_0.1)	
SZ_31682	F	12	Sporadic	*ATM*	CmpHET	TRUE	NM_000051.4:c.5697C>A:p.Cys1899*	0	NA/NA/NA/NA/B	.	Known	P	P (PVS1, PM2, PM3, PM5)	*Probable*
						TRUE	NM_000051.4:c.8161G>A:p.Asp2721Asn	0	D/D/D/U/B		Known	Conflict	VUS (PM2, PM3, PP3)	
SZ_31719	F	17	Sporadic	*SETX*	CmpHET	TRUE	NM_015046.7:c.6843-4_6843-1delinsT	NA	NA/NA/NA/NA/NA	.	Novel	Novel	LP (PVS1, PM2)	*Definitive*
						TRUE	NM_015046.7:c.129T>G:p.Cys43Trp	NA	D/D/D/D/B		Novel	Novel	LP (PM2, PM3, PP3)	
SZ_32512	M	13	Sporadic	*PMPCA*	HOM	TRUE	NM_015160.3:c.524G>C:p.Arg175Pro	1.1E-05	B/U/D/B/B	.	Known	Novel	VUS (PM2)	*Unclear*
SZ_32703	F	29	AD	*KCNC3*	HET	NA	NM_004977.3:c.1268G>A:p.Arg423His	0	D/D/D/D/B	.	Known	P	P (PM1, PM2, PM5, PP1, PP3, PS2, PS3)	*Definitive*
SZ_32920	F	27	Sporadic	*STUB1*	CmpHET	TRUE	NM_005861.4:c.433A>C:p.Lys145Gln	5.4E-04	D/B/D/D/B	.	Known	Conflict	LP (PM1, PM2, PM3, PP2, PP3)	*Definitive*
						TRUE	NM_005861.4:c.689_692del:p.Tyr230Cysfs*9	2E-06	NA/NA/NA/NA/NA		Known	P	P (PVS1, PS4, PM2)	
SZ_32973	M	13	Sporadic	*VPS13D*	CmpHET	TRUE	NM_015378.4:c.448-2A>C	NA	NA/NA/NA/NA/S	.	Novel	Novel	LP (PVS1, PM2)	*Unclear*
						TRUE	NM_015378.4:c.12632T>C:p.Val4211Ala	NA	U/D/D/U/B		Novel	Novel	VUS (PM2, PM3)	

Table S7. Previously unreported genetic findings by our group attributed to conventional and structural variants identified by targeted NGS. (continued)

Patient ID	Sex	AAO (years)	Suspected Inheritance	Gene	Zygosity	Family Segregation	Variant	gnomAD (aggregated)	In Silico Predictions (REVEL/AlphaMissense/VARITY/PrimateAI/SpliceAI)	Other Findings	HGMD	ClinVar	ACMG Classification	Molecular Diagnosis
SZ_32981	M	19	AR (consanguineous)	*SETX*	HOM	NA	NM_015046.7:c.7034_7036del:p.Ile2345del	NA	NA/NA/NA/NA/B	.	Novel	Novel	VUS (PM2, PM4)	*Probable*
SZ_33433	M	19	AD	*PRKCG*	HET	TRUE	NM_002739.5:c.253T>C:p.Cys85Arg	NA	D/D/D/D/B	.	Novel	Novel	VUS (PM1, PM2, PP2, PP3)	*Probable*
SZ_33537	M	4	Sporadic	*ADPRHL2*	CmpHET	TRUE	NM_017825.3:c.400_411del:p.Asp134_Glu137del	1E-06	NA/NA/NA/NA/NA	.	Novel	Novel	VUS (PM2, PM4)	*Unclear*
						TRUE	NM_017825.3:c.307_308delinsCC:p.Arg103Pro	0	NA/NA/NA/NA/NA		Novel	Novel	VUS (PM2)	
SZ_34104	M	30	Sporadic	*SYNE1*	CmpHET	TRUE	NM_182961.4:c.11550T>A:p.Tyr3850*	NA	NA/NA/NA/NA/NA	.	Novel	Novel	LP (PVS1, PM2)	*Definitive*
						TRUE	NM_182961.4:c.10172G>A:p.Tyr3391*	NA	NA/NA/NA/NA/NA		Novel	Novel	LP (PVS1, PM2)	
SZ_34109	F	52	Sporadic	*STUB1*	HET	NA	NM_005861.4:c.691C>G:p.Leu231Val	NA	U/D/D/D/B	*ATXN8* (28/121)	Known	Novel	VUS (PM1, PM2, PP2)	*Unclear*
SZ_34686	F	46	Sporadic	*SACS*	CmpHET	TRUE	NM_014363.6:c.171+2T>A	0	NA/NA/NA/NA/S	.	Novel	Novel	LP (PVS1, PM2)	*Unclear*
						TRUE	NM_014363.6:c.13720T>G:p.Phe4574Val	1E-06	U/U/D/U/B		Novel	Novel	VUS (PM2, PM3)	
SZ_34726	F	2	AD	*SPTBN2*	HET	TRUE	NM_006946.4:c.1310G>A:p.Arg437Gln	0	U/D/D/D/B	.	Known	P	LP (PS2, PM1, PM2, PM5)	*Definitive*
SZ_35226	M	19	Sporadic	*POLG*	CmpHET	TRUE	NM_002693.3:c.3091G>T:p.Glu1031*	NA	NA/NA/NA/NA/B	.	Novel	Novel	LP (PVS1, PM2)	*Definitive*
						TRUE	NM_002693.3:c.2348C>T:p.Pro783Leu	NA	U/B/B/U/B		Novel	Novel	LP (PM1, PM2, PM3, PP2)	
SZ_35360	F	49	AD	*PRKCG*	HET	NA	NM_002739.5:c.466G>A:p.Glu156Lys	NA	U/D/D/D/B	.	Known	Conflict	VUS (PM2, PP2)	*Unclear*
SZ_35539	F	21	Sporadic	*STUB1*	CmpHET	TRUE	NM_005861.4:c.865G>A:p.Val289Ile	8E-06	B/U/B/U/B	.	Known	Novel	VUS (PM2, PP2)	*Unclear*
						TRUE	NM_005861.4:c.853G>A:p.Ala285Thr	NA	U/D/D/D/B		Novel	VUS	VUS (PM2, PP2)	
SZ_35781	M	9	Sporadic	*CWF19L1*	CmpHET	TRUE	NM_018294.6:c.1555_1557del:p.Glu519del	4.3E-05	NA/NA/NA/NA/B	.	Novel	VUS	VUS (PM2, PM4)	*Unclear*
						TRUE	NM_018294.6:c.1070G>T:p.Gly357Val	NA	B/U/D/U/B		Known	P	VUS (PM2, PM3)	
SZ_35940	M	26	Sporadic	*SYNE1*	CmpHET	TRUE	NM_182961.4:c.11113_11119del:p.Gln3705Trpfs*32	NA	NA/NA/NA/NA/NA	.	Novel	Novel	LP (PVS1, PM2)	*Definitive*
						TRUE	NM_182961.4:c.13420C>T:p.Arg4474*	1.2E-05	NA/NA/NA/NA/NA		Known	LP	P (PVS1, PS4, PM2)	
SZ_36382	F	22	AR	*SACS*	HOM	NA	NM_014363.6:c.11274_11276del:p.Ile3758_Thr3759delinsMet	1E-06	NA/NA/NA/NA/B	.	Known	Conflict	VUS (PM2, PM4)	*Probable*
SZ_36446	F	2	Sporadic	*SACS*	HOM	NA	NM_014363.6:c.13706_13708del:p.Ile4569del	NA	NA/NA/NA/NA/B	.	Novel	Novel	VUS (PM2, PM4)	*Probable*
SZ_36732	M	46	AR	*SACS*	CmpHET	TRUE	NM_014363.6:c.178del:p.Asp60Thrfs*8	NA	NA/NA/NA/NA/B	.	Novel	Novel	LP (PVS1, PM2)	*Definitive*
						TRUE	NM_014363.6:c.4723C>T:p.Arg1575Trp	4E-06	D/D/B/D/B		Novel	VUS	LP (PM2, PM5, PP3)	
SZ_36876	F	5	Sporadic	*MRE11*	CmpHET	TRUE	NM_005591.4:c.1920T>A:p.Tyr640*	0	NA/NA/NA/NA/B	.	Novel	P	LP (PVS1, PM2)	*Probable*
						TRUE	NC_000011.10:g.94423022_94439328dup	NA	NA/NA/NA/NA/NA		Novel	Novel	VUS (2I_0.45)	

Patient IDs are prefixed with HS or SZ, corresponding to the recruitment medical center HSFU (HS) and SAHZU (SZ), respectively.

ClinVar classifications: P: pathogenic; LP: likely pathogenic; VUS: variant of uncertain significance; Conflict: conflicting classifications of pathogenicity.

AAO: age at onset; AD: autosomal dominant; AR: autosomal recessive; B: benign; D: deleterious; F: female; HGMD: the Human Gene Mutation Database; M (Sex): male; M (In Silico Predictions): moderate; NA: not available; S: strong; U: uncertain.

Figure S1. Molecular characterization of the five probands identified with pathogenic CNVs.

The first proband with *MRE11* as the causative gene was found to carry a NM_005591.4: c.1920T>A: p.Tyr640* variant and an additional duplication in the same gene. WGS confirmed the duplication as a 16.3-kb repeat at chr11:94,423,022–94,439,328 (hg38). Familial segregation analysis was consistent with an AR inheritance pattern. The second individual with genetically confirmed oculomotor apraxia type 2 was found to harbor both a NM_015046.7: c.6695G>A: p.Arg2232His variant and a heterozygous intragenic deletion in *SETX*. WGS and Sanger sequencing localized the deletion to chr9:132,325,334–132,350,327 (hg38), spanning approximately 25 kb. Inheritance pattern analysis within the family supported AR transmission, with both variants segregating in trans. In the third case, WGS confirmed a roughly 10-kb homozygous intragenic deletion within *SETX* at chr9:132,331,125–132,341,167 (hg38). Subsequent Sanger sequencing showed a single adenine insertion in the deleted region. However, the segregation analysis was not available. The fourth patient diagnosed with *SPG7*-related ataxia was identified to carry a NM_003119.4: c.637C>T: p.Arg213* variant alongside a genomic deletion. The deletion, initially verified by multiplex ligation-dependent probe amplification (MLPA), was further refined by Sanger sequencing of the flanking PCR amplicon, which defined the breakpoints at chr16:89,532,845–89,549,420 (hg38). To ascertain the phase of these two variants in the absence of parental DNA, an allele-specific long-range PCR strategy was employed, using primers flanking the nonsense variant and the deletion's SNV-proximal breakpoint. Subsequent Sanger sequencing of the PCR products confirmed that the two variants were in a trans configuration, a finding consistent with an AR transmission. The fifth patient, also diagnosed with *SPG7*-related ataxia, was found to carry a NM_003119.4: c.2246del: p.Pro749Argfs*9 variant alongside a large genomic deletion at chr16:89,505,632 – 89,538,006delinsT, spanning approximately 32.4 kb and encompassing multiple exons of *SPG7*. The deletion was initially identified by ExomeDepth-based CNV analysis, subsequently verified by MLPA, and further refined by Sanger sequencing of the breakpoint-flanking PCR amplicons. Familial segregation analysis confirmed that the two variants were in a trans configuration, consistent with compound heterozygosity and AR transmission.

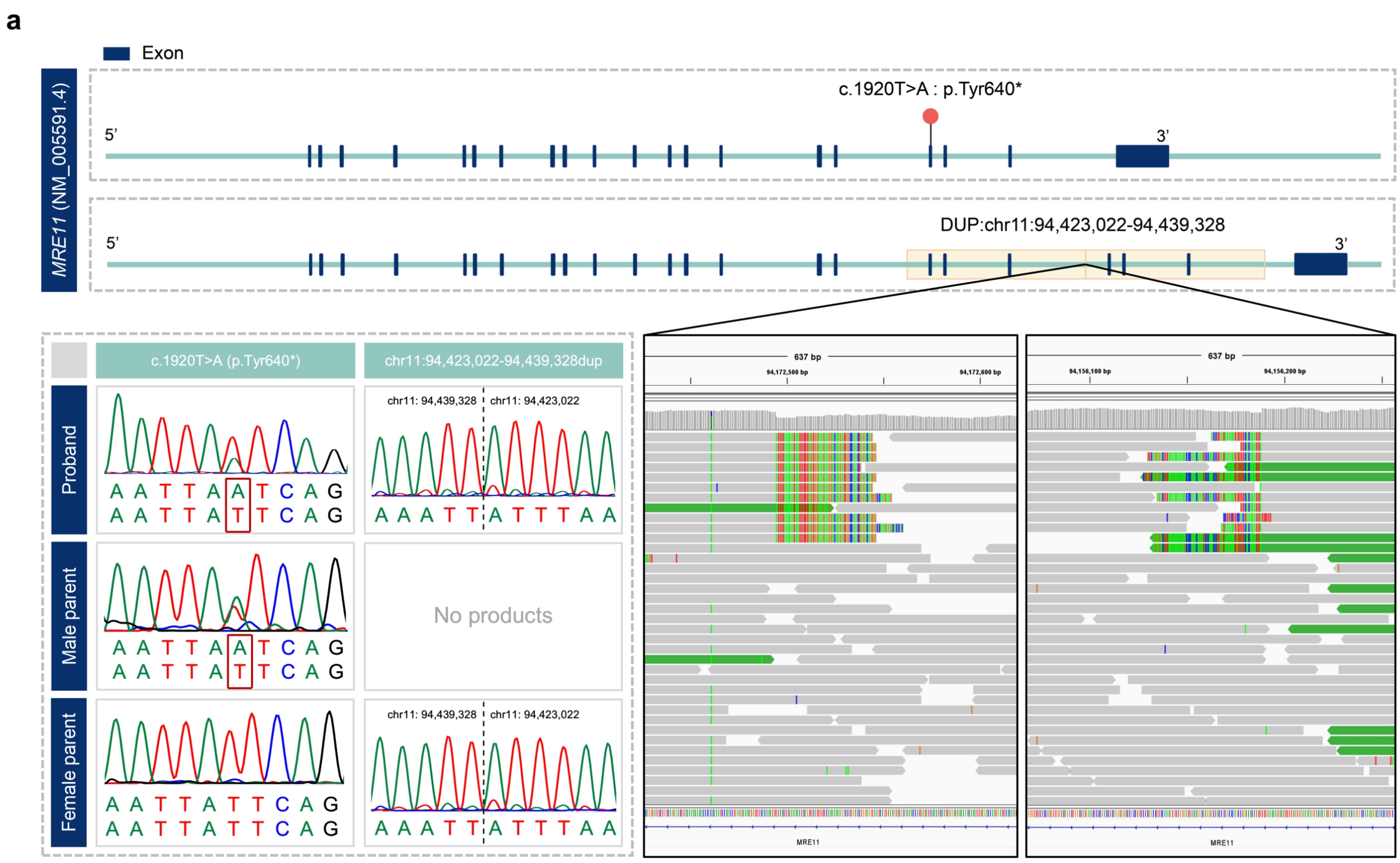

b

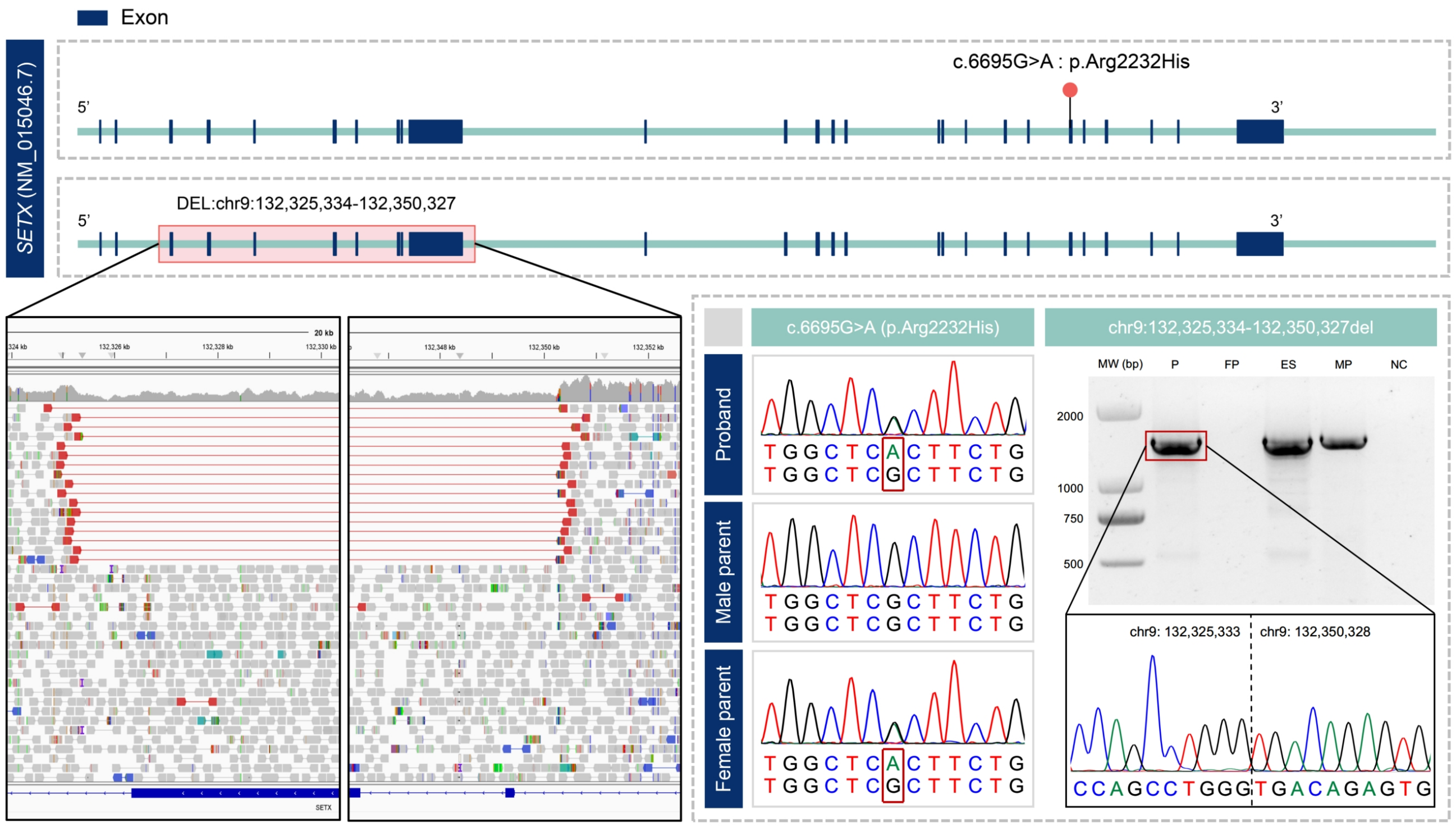
Exon
SETX (NM_015046.7)
c.6695G>A : p.Arg2232His
5'
3'
DEL:chr9:132,325,334-132,350,327
c.6695G>A (p.Arg2232His)
chr9:132,325,334-132,350,327del
Proband
Male parent
Female parent
TGGCTCACTTCTG
TGGCTCGCTTCTG
MW (bp)
P
FP
ES
MP
NC
2000
1000
750
500
chr9: 132,325,333
chr9: 132,350,328
CCAGCCTGGGTGACAGAGTG

c

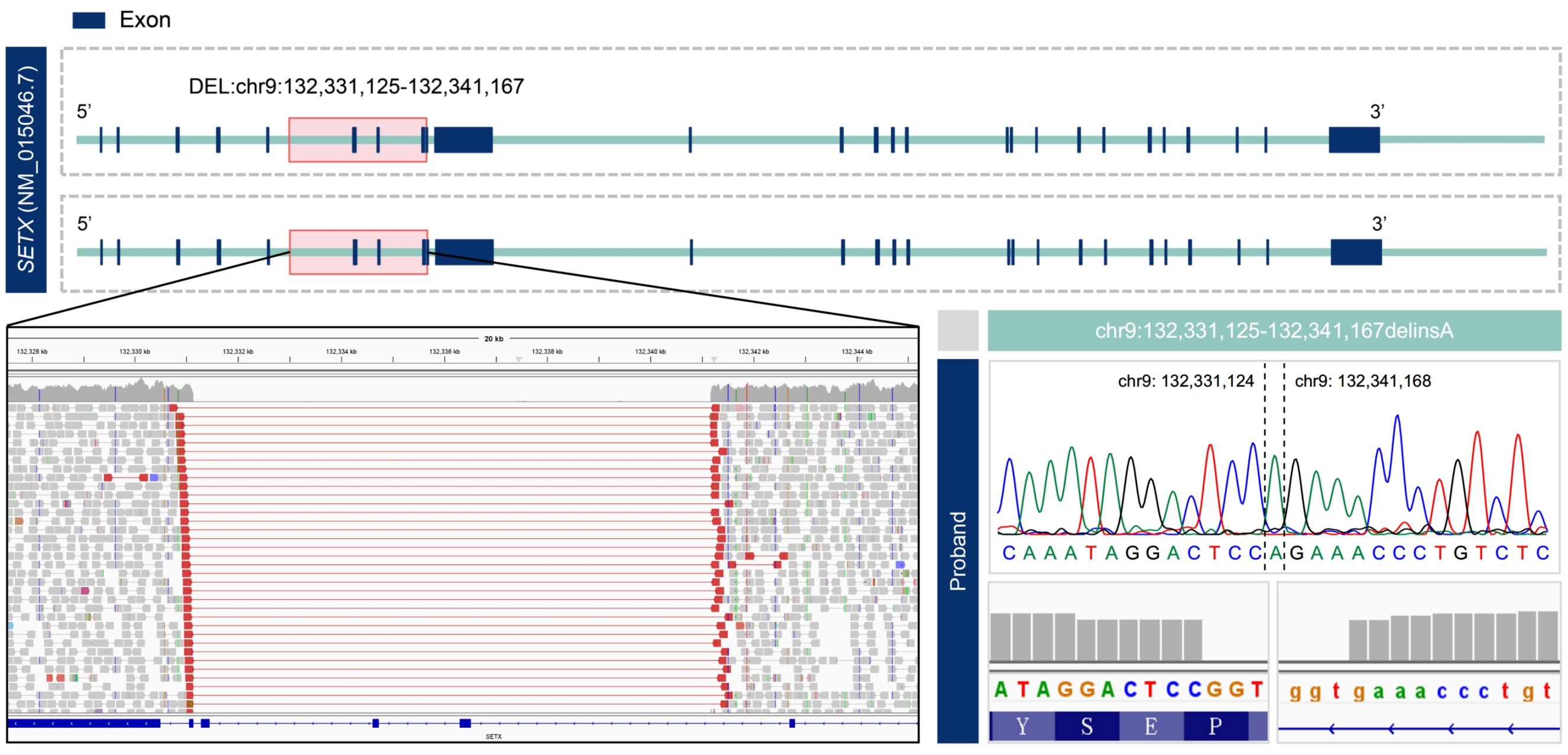
Exon
SETX (NM_015046.7)
DEL:chr9:132,331,125-132,341,167
5'
3'
chr9:132,331,125-132,341,167delinsA
Proband
chr9: 132,331,124
chr9: 132,341,168
CAAATAGGACTCCAGAAACCCTGTCTC
ATAGGACTCCGGT
ggtgaaaccctgt
Y S E P

d

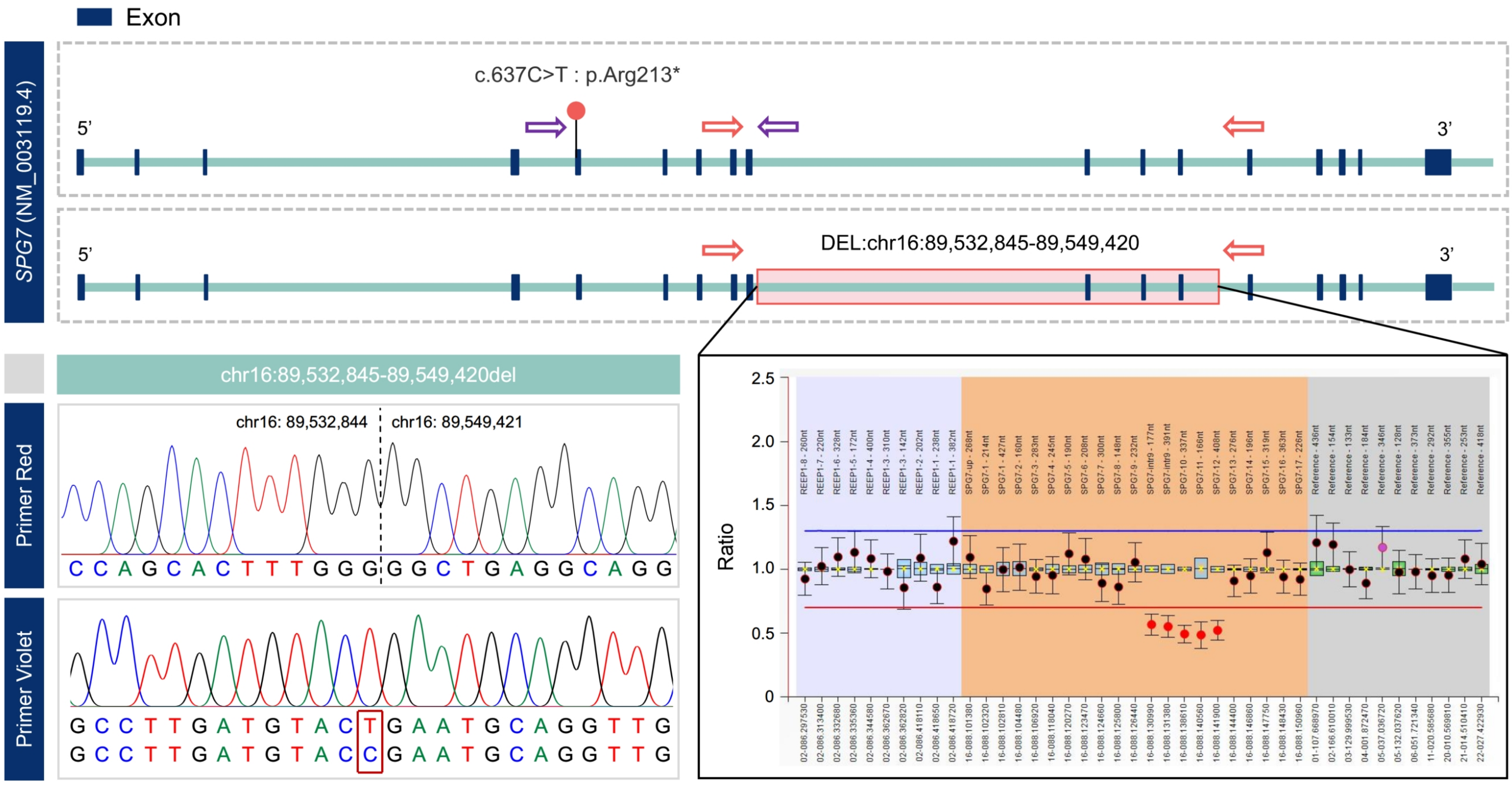

e

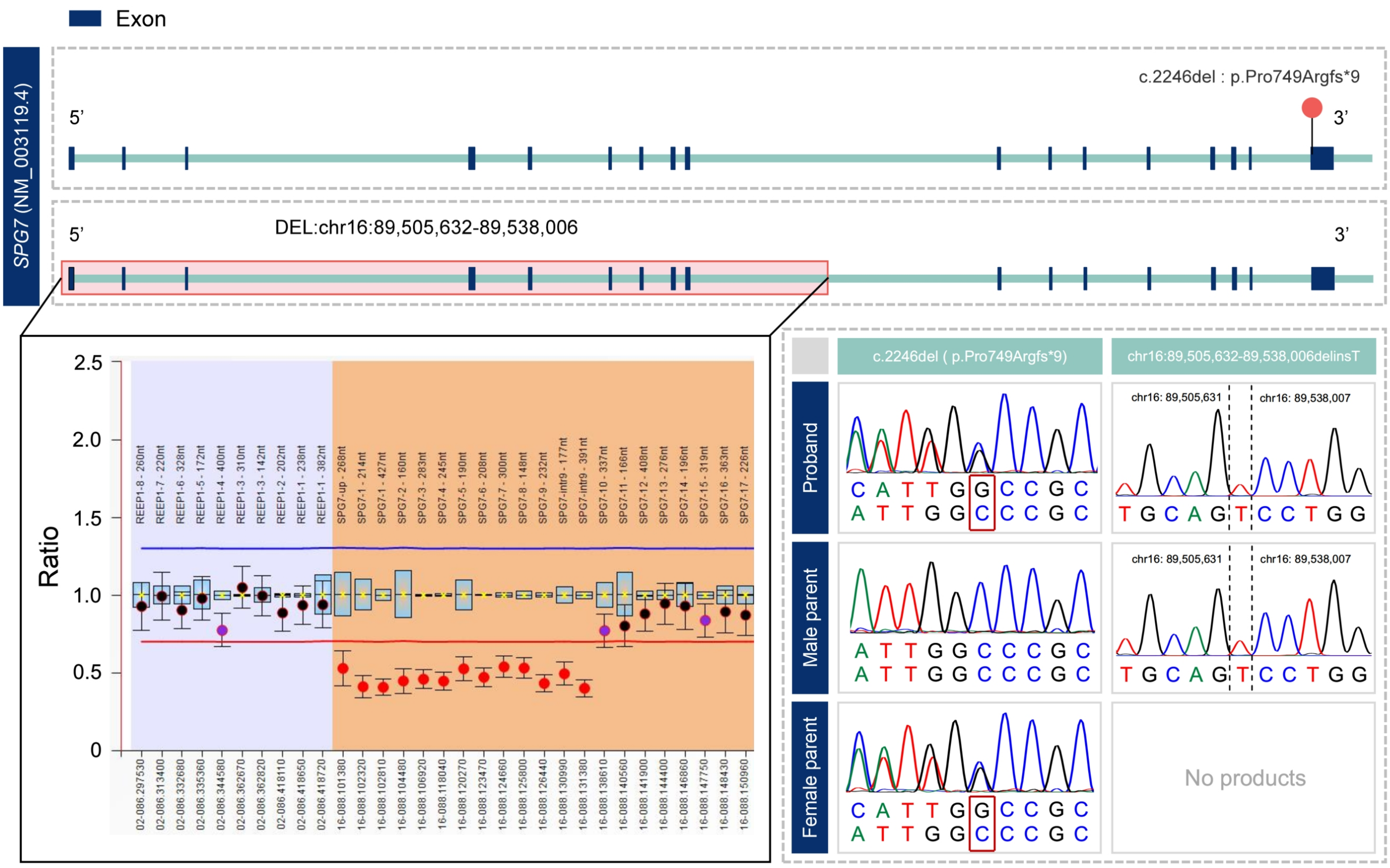

The causative variant profiles of the five probands carrying pathogenic CNVs in *MRE11* **(a)**, *SETX* **(b and c)**, and *SPG7* **(d and e)** are shown. Four of them were compound heterozygous for both a pathogenic SNV and an allelic CNV. The CNVs are detailed by the Integrative Genomics Viewer plots of PCR-free short-read WGS data or by the results of MLPA. PCR products spanning the breakpoints were subjected to Sanger sequencing for further molecular characterization. The allelic phase was established in all compound heterozygous cases. Particularly, for the fourth case, an allele-specific long-range PCR strategy was performed on high-molecular-weight genomic DNA, using primers flanking the nonsense variant and the deletion's SNV-proximal

breakpoint (primer violet). Subsequent Sanger sequencing of the amplicon revealed that the SNV and CNV were not in cis.

ES: elder sister; FP: female parent; MLPA: multiplex ligation-dependent probe amplification; MP: male parent; MW: molecular weight; NC: negative control; P: proband.

Table S8. Aligned genotype distribution of autosomal dominant ataxia between Chen et al. and the HARMONY cohort.

Genotype	Chen et al. (n)	Chen et al. Proportion (%)	HARMONY (n)	HARMONY Proportion (%)
ATXN3	693	73.9%	1249	71.9%
ATXN2	102	10.9%	150	8.6%
ATXN1	81	8.6%	139	8.0%
CAG-*CACNA1A*	22	2.3%	51	2.9%
ATXN7	12	1.3%	20	1.2%
NOP56	9	1.0%	36	2.1%
ATXN8	6	0.6%	15	0.9%
TBP	4	0.4%	6	0.3%
PPP2R2B	2	0.2%	35	2.0%
TGM6	2	0.2%	0	0.0%
ATN1	1	0.1%	8	0.5%
PRKCG	1	0.1%	3	0.2%
CACNA1A	1	0.1%	9	0.5%
TMEM240	1	0.1%	2	0.1%
CCDC88C	1	0.1%	0	0.0%
SPTBN2	0	0.0%	2	0.1%
TTBK2	0	0.0%	0	0.0%
KCNC3	0	0.0%	3	0.2%
ITPR1	0	0.0%	1	0.1%
KCND3	0	0.0%	2	0.1%
PDYN	0	0.0%	0	0.0%
FGF14	0	0.0%	0	0.0%
AFG3L2	0	0.0%	3	0.2%
ELOVL4	0	0.0%	0	0.0%
ELOVL5	0	0.0%	0	0.0%
CACNA1G	0	0.0%	1	0.1%
ATXN10	0	0.0%	2	0.1%
BEAN1	0	0.0%	0	0.0%
**Total**	938	100.0%	1737	100.0%

Table S9. Aligned genotype distribution of ataxia between Wan et al. and the HARMONY cohort.

Genotype	Wan et al. (n)	Wan et al. Proportion (%)	HARMONY (n)	HARMONY Proportion (%)
ATXN3	104	57.8%	1249	69.2%
ATXN2	18	10.0%	150	8.3%
ATXN1	17	9.4%	139	7.7%
ATXN3;*TBP*-i	5	2.8%	1	0.1%
CAG-*CACNA1A*	4	2.2%	51	2.8%
CCDC88C	3	1.7%	0	0.0%
SPG7	3	1.7%	5	0.3%
ATXN8	3	1.7%	16	0.9%
SYNE1	3	1.7%	11	0.6%
ATXN3;*PPP2R2B*	2	1.1%	1	0.1%
CACNA1G	2	1.1%	1	0.1%
SPTBN2	2	1.1%	2	0.1%
TBP	2	1.1%	21	1.2%
SACS	2	1.1%	17	0.9%
PPP2R2B	1	0.6%	35	1.9%
CYP7B1	1	0.6%	0	0.0%
TGM6	1	0.6%	0	0.0%
TTBK2	1	0.6%	0	0.0%
POLR3A	1	0.6%	1	0.1%
ANO10	1	0.6%	1	0.1%
POLG	1	0.6%	1	0.1%
PRKCG	1	0.6%	4	0.2%
SETX	1	0.6%	14	0.8%
ATN1	1	0.6%	8	0.4%
ATXN7	0	0.0%	20	1.1%
CACNA1A	0	0.0%	9	0.5%
COX20	0	0.0%	9	0.5%
ATXN3;*ATXN2*	0	0.0%	5	0.3%
ATM	0	0.0%	4	0.2%
AFG3L2	0	0.0%	3	0.2%
KCNC3	0	0.0%	3	0.2%
ATXN10	0	0.0%	2	0.1%
STUB1	0	0.0%	3	0.2%
ATXN2;*TBP*-i	0	0.0%	2	0.1%
ITPR1	0	0.0%	2	0.1%
KCND3	0	0.0%	2	0.1%
TMEM240	0	0.0%	2	0.1%
TTPA	0	0.0%	2	0.1%
ADCK3	0	0.0%	1	0.1%
APTX	0	0.0%	1	0.1%
ADPRHL2	0	0.0%	1	0.1%
ATXN1;*TBP*-i	0	0.0%	1	0.1%

Table S9. Aligned genotype distribution of ataxia between Wan et al. and the HARMONY cohort. (continued)

Genotype	Wan et al. (n)	Wan et al. Proportion (%)	HARMONY (n)	HARMONY Proportion (%)
CWF19L1	0	0.0%	1	0.1%
GDAP2	0	0.0%	1	0.1%
KIF1C	0	0.0%	1	0.1%
MRE11	0	0.0%	1	0.1%
**Total**	180	100.0%	1804	100.0%
